# Exploring geothermal energy prospects through radioelement enrichment zones in Gabal Abu Hashim area in southeastern Aswan by geophysical and remote sensing data integration

**DOI:** 10.1038/s41598-024-77393-8

**Published:** 2024-11-07

**Authors:** Shaimaa M. El-Hadidy, Mohamed A. S. Youssef

**Affiliations:** 1 Geology Department, Faculty of Science, Ain Shams University, Cairo, Egypt; 2 Exploration Division, Nuclear Materials Authority, Cairo, Egypt

**Keywords:** Remote sensing, Alteration zones, AGRS, Radiogenic heat production, Gabal Abu Hashim GIS modeling, Environmental sciences, Natural hazards

## Abstract

Alteration zones in the Eastern Desert are promising for minerals and geological resources exploration. Remote sensing and geophysical techniques offer cost-effective tools for identifying new exploration sites. Additionally, their use in mapping potential alteration zones is crucial for enhancing exploration. Geophysical and remote sensing data are integrated to perform a comprehensive study of minerals alteration associated with radioactive materials under controlling geological structures. This study aims to explore the associated radiogenic heat and geothermal energy to expand the geothermal resources assessments. The study utilizes Spectral Angle Mapper classification, band algebra, principal component analysis (PCA), surface lineament density, and decorrelation stretch techniques and Multiple Criteria Decision Analysis to enhance the mapping of mineralized alterations in the study area. It uses VNIR-SWIR ASTER data to identify hydrothermal alteration minerals and dominant alteration zones, also Landsat 8 Thermal Infrared Sensor (TIRS) offers two adjacent thermal bands, benefiting land surface temperature (LST) radiation from space in the Gabal Abu Hashim area. The area contains minerals alteration indicators like chlorite, alunite, illite, kaolinite, sericite, with less abundant ferrous minerals and epidote. Airborne gamma-ray spectrometry (AGRS) was used to identify naturally occurring radioactive anomalous zones, including potassium (K), equivalent uranium (eU), and equivalent thorium (eTh), to estimate the radiogenic heat production (RHP) in selected areas of the study area. The examination of AGRS data indicated that the studied region has radioelement concentrations ranging from 2.8 to 148 ppm, 18 to 144 ppm, and 0.004 to 9% of (eU), (eTh), and (K), respectively, indicating the existence of various rock types. The Radioelements Composite Image (RCI) successfully highlighted the radioelements enriched zones associated with younger granite, older granite, and metamorphic rocks, particularly those with extensive hydrothermal alteration. The results successfully discriminated alteration zones associated with radioelements K, U, and Th potential parts in the regional shear oblique zone. The weighted overlay GIS model was used to produce the alteration zones potentiality map, and to identify five zones of significant variations in heat production across different geological formations. The northern and southeastern regions demonstrate high alterations and land surface temperature corresponding to areas of high fault density and shear zones. The results of this study reveal that the proposed methods of remote sensing and AGRS data are effective in detecting areas rich in K, eU, and eTh in alteration zones associated with high radiogenic heat production in younger granite, older granite, and metamorphic rocks.

## Introduction

Radiogenic heat production, a significant source of heat in Earth’s crust, is generated by decaying radioactive isotopes as uranium, thorium, and potassium. It drives geological activities, contributes to geothermal energy, influences Earth’s crust’s internal temperature and structure, affects heat flow, potentially impacts ecosystems and reduces reliance on fossil fuels^1^.

Geophysical methods like gamma-ray spectrometry can measure the radioelements concentrations in rocks, revealing variations across different geological formations, as seen in many global studies such as Western Himalaya studies^2^. High concentrations of radioelements in areas like the Sierras de Córdoba in Argentina are prime targets for geothermal energy exploration due to their elevated gradients^1^.

Hydrothermal alteration mineral assemblages like illite, montmorillonite, chlorite, kaolinite, apatite, scarbro, and albite indicate geothermal energy-associated eruptions in terrestrial and extraterrestrial bodies. Distribution and character are influenced by temperature, heat, and chemical diffusion. Hydrothermal alteration minerals and their zonation indicate potential geothermal energy-associated zones^3^. To ensure success, integrated geological, geophysical, remote sensing, and hydrogeochemical Studies were proposed to delineate geothermal prospects and explore zones of enrichment of radioactive elements. However, advancements in remote sensing technology and digital image processing have enabled mapping of hydrothermal minerals and alteration zones, reducing exploration time and cost in the early stages.

Remote sensing technologies like satellite imagery and aerial surveys aid in mapping surface alterations, identifying geothermal zones, and detecting anomalies in surface temperature and mineral composition. The Advanced Spaceborne Thermal Emission and Reflection Radiometer (ASTER) is a state-of-the-art instrument sensor system on the Terra spacecraft, launched in 1999. It offers wide spectral coverage and high spatial resolution in the visible near-infrared, shortwave infrared, and thermal infrared regions. ASTER data is used in various global change-related applications, including vegetation and ecosystem dynamics, hazard monitoring, geology, soils, hydrology, and land cover change, https://lpdaac.usgs.gov. ASTER, an advanced spaceborne thermal emission and reflection radiometer, is widely used for mapping hydrothermal alteration minerals and alteration zones associated with geothermal resources, with 14 bands in visible, near-infrared, SWIR, and TIR regions. The identification of alteration zones with minerals like argillic-phyllic1 is achieved using techniques like color composite, band ratios, and Relative Band Depth (RBD),^4^. ASTER data has been used in studies to identify hydrothermal alteration minerals and alteration zones for mineral and geothermal resource exploration,^4–6^ used day and nighttime data to map geothermal and mineral mapping in East Africa. Remote thermal sensors are crucial in soil sciences due to their ability to measure emissivity, which determines radiation emission at a specific temperature. High emissivities are common for most materials, except for metals and minerals. Thermal remote sensing (3 to 15 μm) region determines the quantity of radiation emitted used for mapping evaporite minerals and hydrothermally altered rocks^3^.

Gamma-ray spectrometry is used in geophysical studies to measure radioelement concentrations in rocks, revealing significant variations in heat production across different geological formations in the study area. The integration of geophysical data and remote sensing information enhances the accuracy of geothermal resource assessments and aids in identifying new geothermal prospects. Geophysical and remote sensing datasets are complex and require robust data management and processing capabilities. Effective integration requires interdisciplinary knowledge in geophysics and remote sensing, which can be challenging to acquire. Accurate calibration and validation of remote sensing data can be time-consuming and costly. Specialized software and tools are often required for integration, which may have steep learning curves and require significant investment in training and resources^8^.

### Study area

**Fig. 1:**
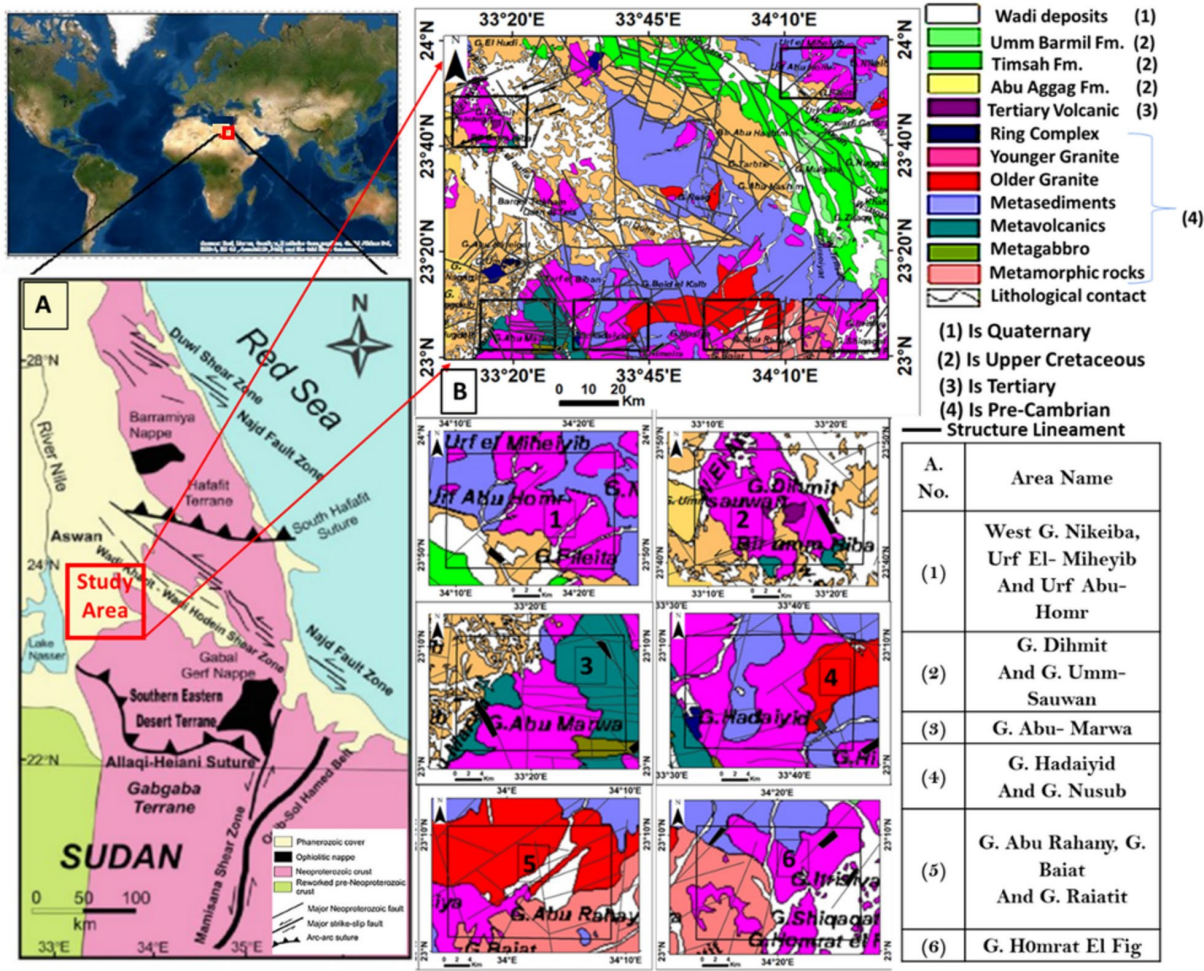
(**A**) Location map of the study area. (**B**) Regional Geologic map of Gabal Abu Hashim area and its surrounding areas, SED, Egypt, (after Conoco Inc. 1987), with Sites 1 to 6 are chosen for detailed analysis based on their detailed surface geology and associated structures.

Gabal Abu Hashim and its surrounding areas are located on the eastern bank of the Nile Valley, Southeastern Desert (SED) of Egypt, (Fig. 1), it is bounded by Latitudes 23° 00′ 00″ and 24° 00′ 00″ N, and longitudes 33° 00′ 00′ and 34° 30′ 00″ E, (Fig. 1). The Eastern Desert has been subjected to multiple tectonic movements, magmatic intrusions and hydrothermal alteration. Many rocks overlaps were formed because of the mountainous movements, forming a complex structural system. These different rock formations and overlaps are linked to the presence of many mineral assemblages of important economic value that attract researchers and investors.

Landsat 8 and ASTER remote sensing data have been utilized to improve the identification of basement rocks in the Eastern Desert of Egypt. Some studies have employed these data for the detection of mineralized alteration zones^9–12,12–18^.

In this study, remote sensing data is primarily used for providing fundamental geology information , geological mapping which is potentially relevant to some aspects of uranium, thorium and potassium mineral exploration, . To better understanding the relationship between RHP and highly possibly alteration zones, remote sensing techniques are being used.. The qualitative and quantitative processes of the remote sensing and AGRS data considering the lithology and structure feature patterns of the surveyed area are used to detect zones that have been related to extensive hydrothermal alteration.

AGRS is extensively used for detailed lithological identification/mapping as well as the detection of radioactive minerals. In addition, it is used in various environmental studies to estimate several radon hazards^9,12,19–23,23–25^ .

The present study aims to recognize and identify the contact zones between the different lithologic units according to different radioactivity levels. The integrated data of enhanced satellite images are used to define zones that have extensive hydrothermal alterations and estimate radiogenic heat production of different geological rock units of the published Conoco geological map of the study area.. Moreover, to throw light on the six highly radio-spectrometric areas and high RHP zones that may be related to these lithological contacts or associated rock units, using the extracted analysis of RCI and the analysis of satellite data

#### Geologic setting

The study area is generally covered by igneous, metamorphic and sedimentary rocks. The northeastern, northwestern, and southern parts of the study area are characterized by Precambrian rocks of metavolcanics, metasediments, and a ring complex, intruded the older and younger granites. About 16 percent of the Eastern Desert basement is made up of younger granites^26,27^ comprising mica granite, alkaline granite, and syenite, and are post-tectonic alkaline to peralkaline rocks with a potassic, sub alkaline to peraluminous biotite granite as the main phase,^28^. Sedimentary rocks (pre-Cretaceous) cover the central and eastern parts of the study area and some localities in northwestern parts. These rocks are represented by Um Barmil, Timsah, and Abu Aggag formations, which contain a proportion of radioactive materials. while the Tertiary volcanic rocks cover the northwestern part and high and low-grade metamorphic rocks are predominantly found in the southeastern part of the study area (Fig.1). Gneissic and migmatitic rocks inhabit substantially bigger, more complexly structured regions connected with batholiths of foliated granodiorite in the South-eastern Desert^29,30^. Geomorphologically, the study area comprises low to medium hills ranging from 200 to 710 meters high, with some high rugged mountains (Fig. 1). Structurally, the area has experienced multiple tectonic events and stresses, resulting in complex deformed structures. It is dissected by different types of faults and fractures of various directions. Some of these faults are primary, while others are secondary. The main fault directions in the Shaait-Nugrus shear zone, as identified by^31^ include NE–SW, NW–SE, WNW–ESE, and ENE–WSW. This shear zone dips north of the line between the Central and South Eastern Desert. Fold-thrust belts dominate the SED, with thrusts representing first-order kinematics that are eventually overprinted by map-scale transgression. The main structures in Southern Egypt, as summarized by^32,33^ include the Gebel Uweinate-Aswan uplift associated with alkaline magma intrusion of NE (Guinean-Nubian lineament) trend during the Triassic-Jurassic and Cretaceous-Tertiary times, E-W faults in the Late Cretaceous and Tertiary times, the NNW Red Sea Trend which marks the onset of the rifting phase during the Turonian or Coniacian, the NW Najd-Fault-System Trend that occurred during the closing time of the Pan African Orogeny and continued until the Devonian times, the NW Gulf of Suez Trend referring to the Pan African Orogeny, and the rejuvenation of the NW trend during different stages of the area’s tectonic evolution^31^. The NE Red Sea Trend occurred during the Oligo-Miocene time.

**Six areas were chosen for detailed analysis based on their surface geology and associated structures.** A careful examination of these areas (Fig. 1) reveal the following characteristics:

Area 1 is covered by wadi deposits, younger granite, and metasediment, and exhibits structure trends in the NE-SW and NW-SE directions. Area 2 is covered by wadi deposits, younger granite, and tertiary volcanic rocks, and shows noticeable strike-slip faults with a NE-SW trend and NW-SE normal faults.

Area 3 is covered by metavolcanic, metagabbro, and younger granites, and displays faults with E-W, NNW-SSE, and NW-SE trends. Area 4 is covered by wadi deposits, younger granites, metagabbro, metasediment, older granite, and a ring complex. The structures in this area trend in the NW-SE, NNE-SSW, and E-W directions. Area 5 is covered by younger granites, older granite, wadi deposits, and metamorphic rocks, and exhibits structures trending in the NW-SE, NNW-SSE, and NE-SW directions. Area 6 is covered by younger granites, wadi deposits, and metamorphic rocks with many structures trending in NNE-SSW and NE-SW normal faults, (Figs. 1, 3).

## Materials and methods

**Fig. 2:**
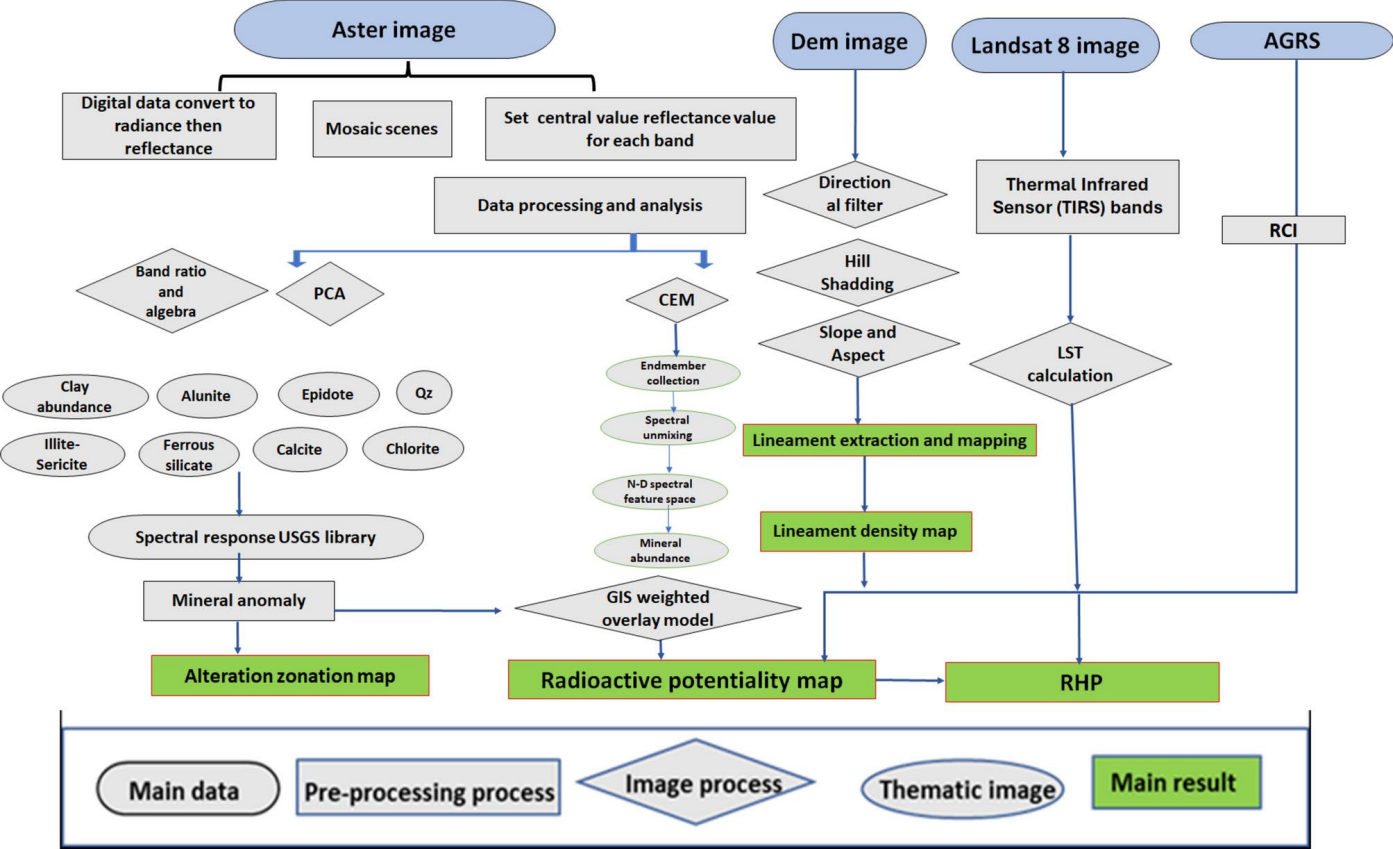
Flow chart clear steps followed in the study.

The ASTER level-1B (L1B) data, Table 1, Landsat 8 data, and airborne gamma-ray spectrometry (AGRS) data (Fig. 2) were used, analyzed and interpreted in this study.

**Table 1:** Parameter and characteristics of Aster sensor and status of different bands. (After https://lpdaac.usgs.gov and https://landsat.gsfc.nasa.gov/).

ASTER				Landsat OLI 8			
Aster bands	central wavelegnth (µm)	Spatial resolution (m)	Swath width (km)	Landsat bands	Spectral range (µm)	Spatial resolution (m)	Swath width (km)
Band 1	VNIR (0.56)	15	60	Band 1	Coastal Aerosol (0.43—0.45 µm)	30	185
Band 2	VNIR (0.66)	15	60	Band 2	Blue (0.450—0.51 µm)	30	185
Band 3	VNIR (0.81)	15	60	Band 3	Green (0.53—0.59 µm)	30	185
Band 4	SWIR (1.65)	30	60	Band 4	Red (0.64—0.67 µm)	30	185
Band 5	SWIR (2.165)	30	60	Band 5	Near-Infrared (0.85—0.88 µm)	30	185
Band 6	SWIR (2.205)	30	60	Band 6	SWIR 1(1.57—1.65 µm)	30	185
Band 7	SWIR (2.26)	30	60	Band 7	SWIR 2 (2.11—2.29 µm)	30	185
Band 8	SWIR (2.33)	30	60	Band 8	(PAN) (0.50—0.68 µm)	15	185
Band 9	SWIR (2.395)	30	60	Band 9	Cirrus (1.36—1.38 µm)	30	185
Band 10	TIR (8.3)	90	60	Band 10	TIRS 1 (10.6—11.19 µm)	100	185
Band 11	TIR (8.65)	90	60	Band 11	TIRS 2 (11.5—12.51 µm)	100	185
Band 12	TIR (9.1)	90	60				
Band 13	TIR (10.6)	90	60

### Remote sensing data

L1B radiance data and Landsat-8 were downloaded from NASA, (http://earthexplorer.usgs.gov), and METIAIST Data Archive System (MADAS). The ASTER data was collected in May 2015 and geocoded to the UTM projection (WGS 84 - Zone 36 N). ASTER data is known for its effectiveness in alteration mapping and mineralogical delineation. The ASTER L1B data which used in this study were radiometrically calibrated and geometrically co-registered. Reflectance and emissivity information of minerals were extracted from the remote sensing data for mineral resources study. The spectral properties of minerals in the ore and host rock were analyzed to determine the effective bands. These created various band ratios and false-color composites to optimize the detection of iron mineralization, alunite, kaolinite, chlorite-epidote assemblage, and argillic alteration.

#### ASTER 1LB preprocessing

Preprocessing techniqueswere applied to the digital ASTER data including mosaic of 13 different scenes into one image, atmospheric correction, and layer stacking of VNIR-SWIR bands. The visible near-infrared (VNIR) and short-wave infrared (SWIR) portions of the spectrum were used for geology analysis due to their ability to capture key absorption features of minerals. ASTER and Landsat 8 images and a Digital Elevation Model (DEM) with 30 m resolution were utilized to visually enhance different rock units and support structural interpretations. VNIR is crucial for detecting iron minerals, while SWIR is effective for clay and carbonate minerals and the TIR range is important for tectosilicate minerals. Spectral mapping of minerals integrated with geological information enabled broad-scale anomaly detection and provided insights into lithology and surface structure.

### Image processing

#### Image restoration

Image restoration was applied to correct inherent defects in the satellite image formed during data collection, such as atmospheric noise. The FLAASH (Fast Line-of-sight Atmospheric Analysis of Spectral Hypercubes) module of the ENVI software was applied to correct the thermal emittance bands of ASTER-TIR and analyze the rapid line-of-sight part of spectral hypercubes. This correction involved converting the radiance at sensor element data to surface reflectance coefficients. The FLAASH technique is an atmospheric correction method that extracts spectral reflectance in the ENVI software. The absorption of certain parts of the electromagnetic spectrum by atmospheric gases was considered. The image restoration process is an important step in extracting valuable information for mineral resources study, including image enhancement and information extraction.

#### Image enhancement

Various enhancement techniques were applied to enhance the information content of the original data:

- **Contrast enhancement:** contrast stretch and a simple linear transformation, were used to increase the contrast of the displayed image by expanding the original grayscale range to fill the lookup table (LUT) of the display device.
- **Spatial filtering:** Directional filters were applied in different directions to enhance linear surface features such as faults, joints, and fractures.
- **Density slicing:** The continual grayscale range was transformed into a series of density intervals (slices), with each slice assigned a separate color.
- **False-color composite image:** A multispectral false-color image was generated by combining different spectral bands and assigning a different color to each band. This technique increased the amount of data available for visual interpretation^34^.

#### Lineament

**Fig. 3:**
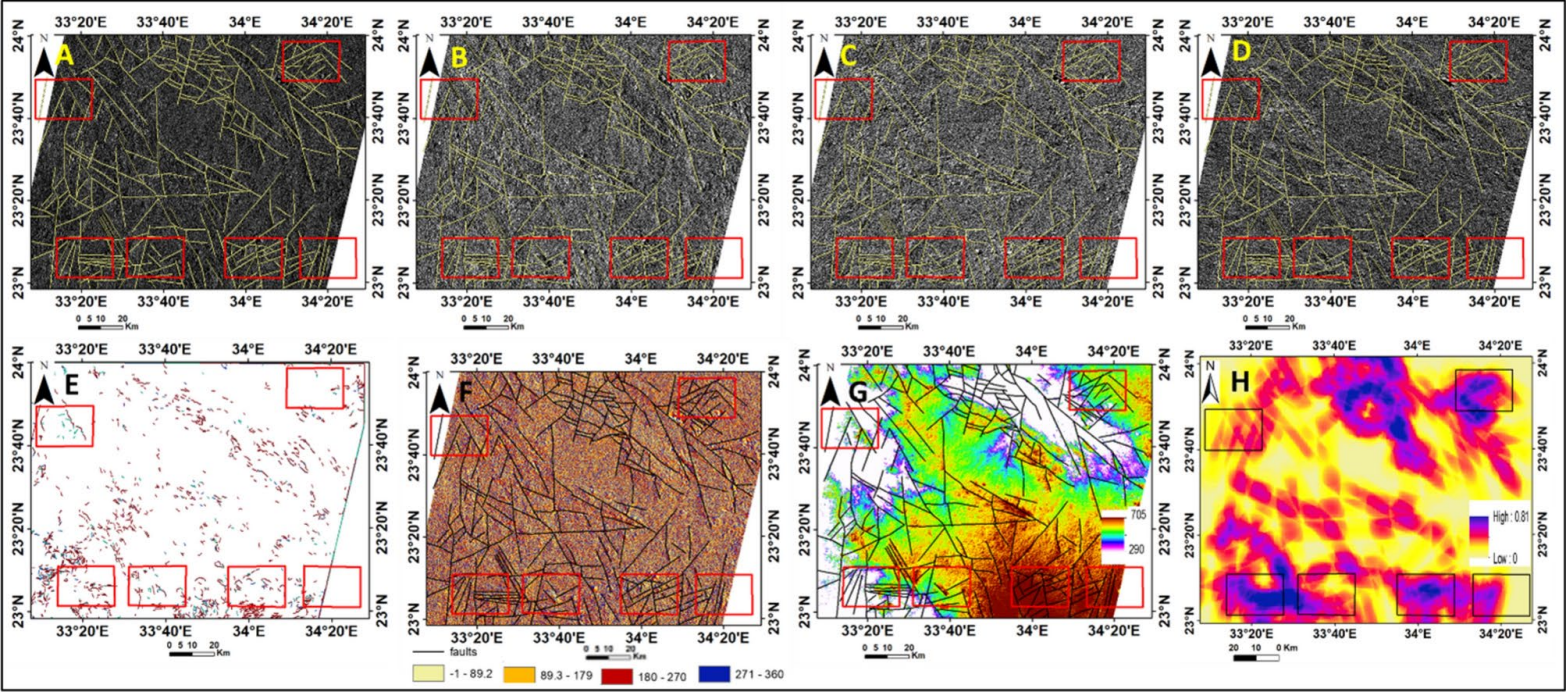
Surface faults and lineament from satellite images. **A**, **B**, **C**, **D** and **F**), The resulting image of a high-pass filter (5*5) with directional Azimuth and Edge filtering, hill shading approach yielded a surface lineament map, (**E**) Automatic extraction of lineaments by Geomatica software, (**G**) the surface lineaments overlaid on the slope from the DEM image and, (**H**) the lineament density map of the study area.

SRTM topographic data (DEM) with a spatial resolution of 30m enabled a detailed interpretation of the structures in the area. Remote sensing techniques such as enhancement, linear and edge filtering, visual interpretation, aspect, and hill shading were applied in various directions (45, 60, 90, 180, and 270 azimuths) using ArcGIS to obtain thematic maps . These maps show the lineament pattern, watershed, lineament density, slope, aspect, and geomorphologic units of the studied area. Maps of slope and aspect were calculated to identify faults (Fig. 3). For example, a steep slope of the terrain is most likely indicate a fault scarp.

- **Lineament density:** Lineament areas were typically extracted and interpreted from satellite imagery manually or automatically. Lineament density refers to the number of linear geological structures on the surface, which are manifestations of underlying geological structures like faults and fractures in each area.^35^ (Fig. 3).

#### Band algebra and band ratio

**Fig. 4:**
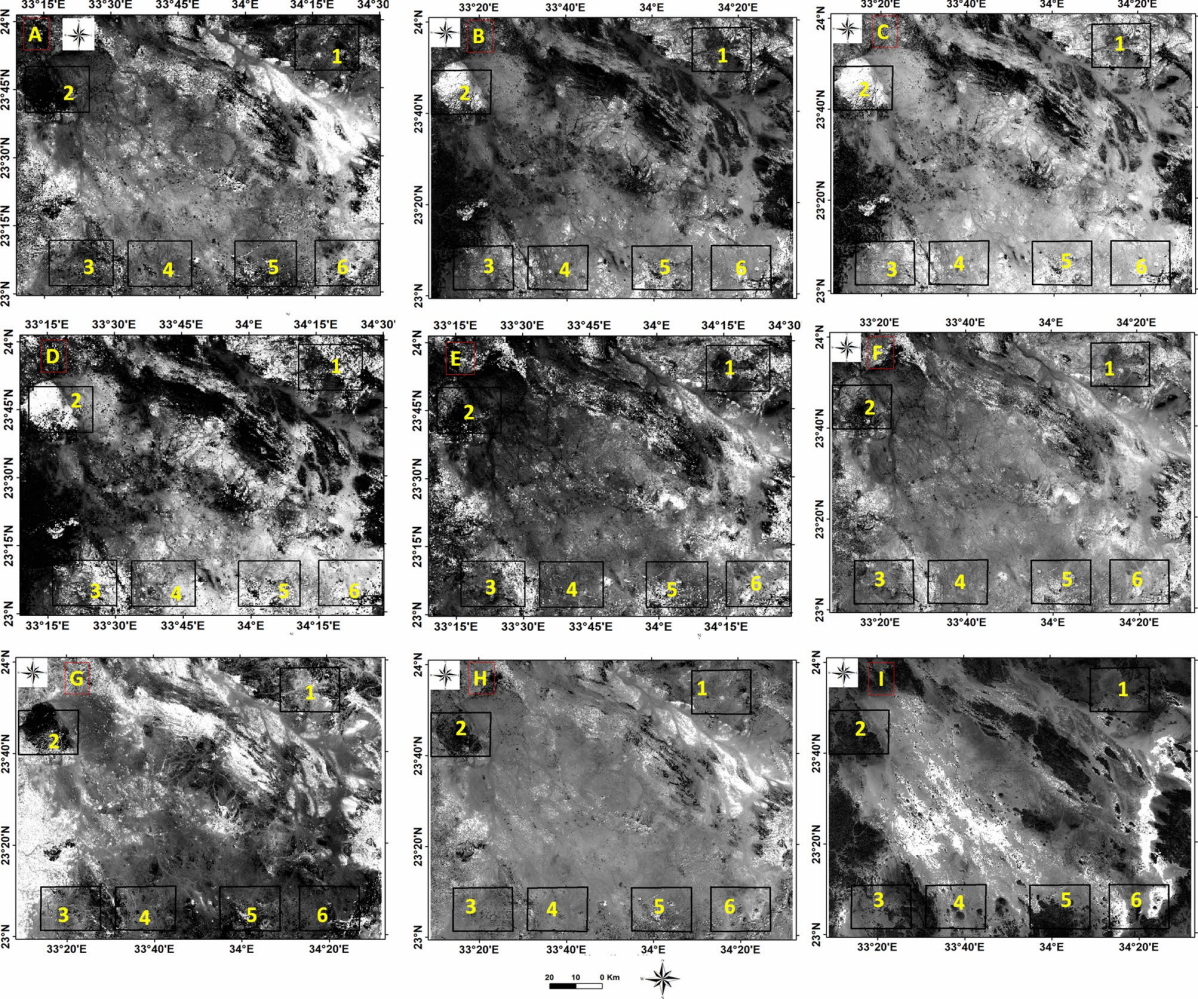
The different band ratios and algebra indices for lithological discrimination and mineralogical distribution outputs. (**A**) Chlorite, (**B**) Sericite- Muscovite, (**C**) Hydroxyl group, (**D**) Quartz, (**E**) Ferrous silicate, (**F**) Argillic- non-argillic, (**G**) clay, (**H**) Illite, and (**I**) Epidote of Gabal Abu Hashim area and its surrounding areas, SED, Egypt.

**Fig. 5:**
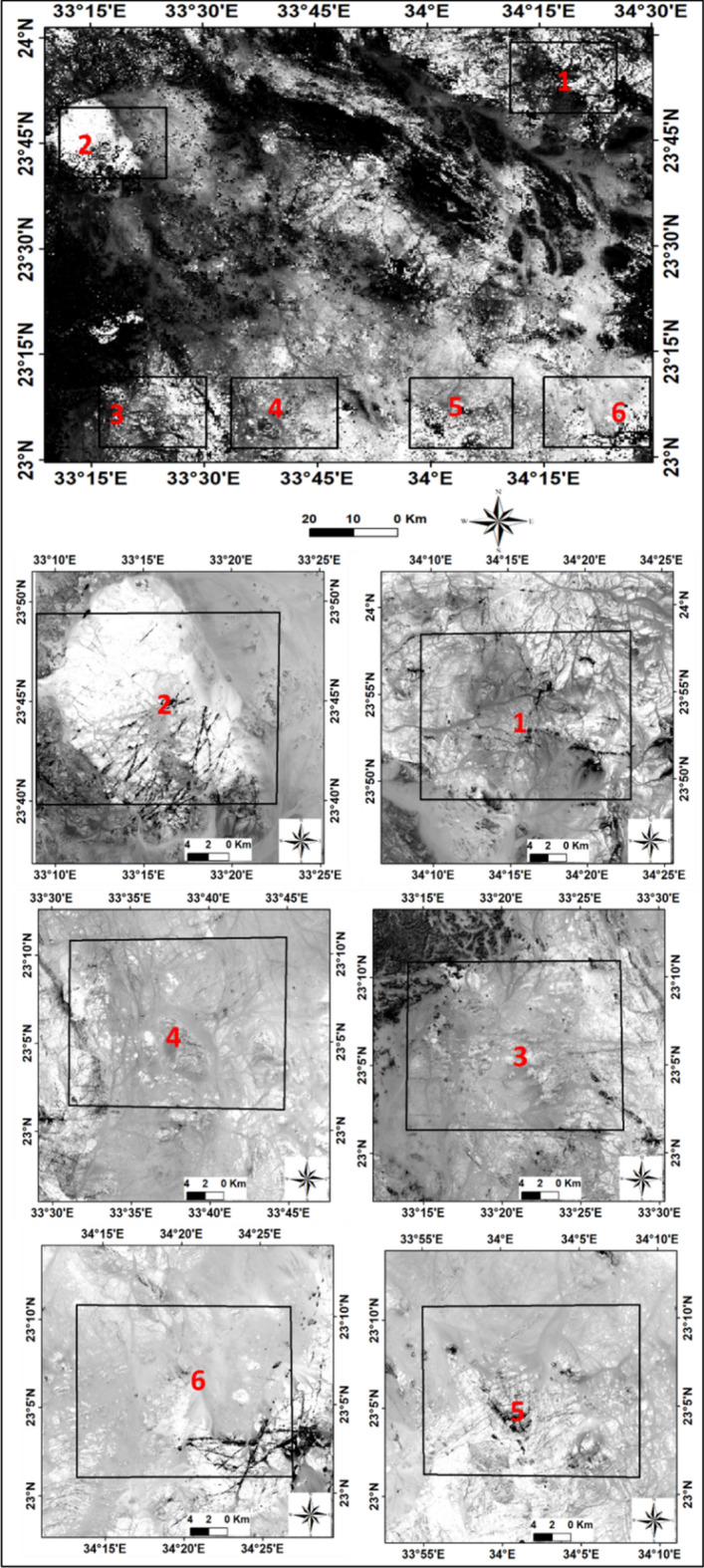
Hydroxyl group marked by white color defining the alteration zones obtained from (b7*b4/b6*b6) band math calculation of Gabal Abu Hashim area and its surrounding areas, SED, Egypt.

**Fig. 6:**
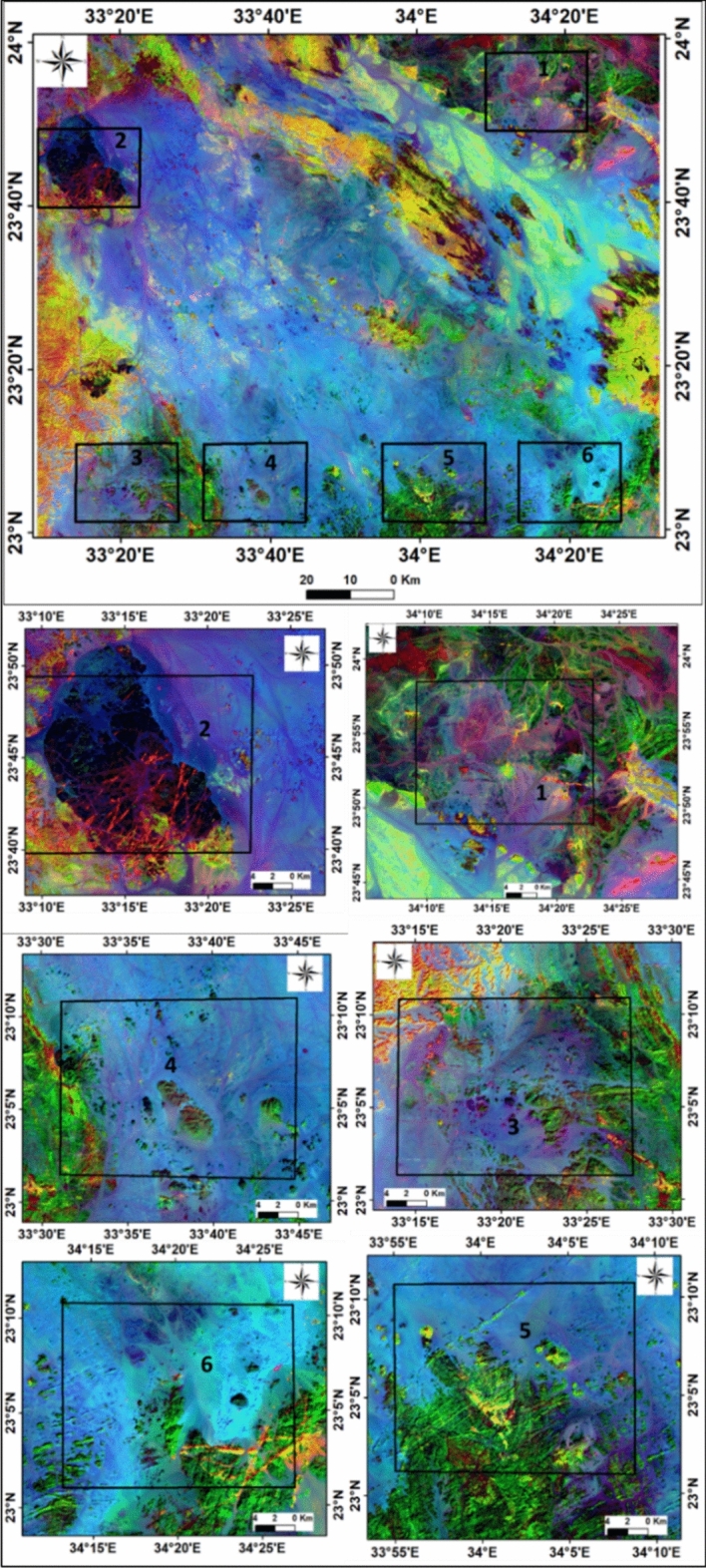
Landsat8 band ratio images. Ratio (b5/b7, b3/b5, b3/b1 RGB) band enlarged the selected areas.

Remote sensing applications in mineral exploration utilize arithmetic equations to analyze different bands and distinguish important minerals from other bedrock. This is performed by dividing the grey level of a pixel in one band by that in another band. Ratios help identify and discriminate valuable minerals from the surrounding parent rock^36^ . By using band ratios in ASTER data, the spectral characteristics related to alteration can be detected. ASTER has even finer spectral bands that enhance alteration discrimination and enable the detection of alteration zones. A complete list of different band ratios (ASTER) used for the identification of various minerals is provided in Table 2, (Figs. 4 and 5). The Landsat 8 ratio (5/7, 3/1, 3/5 RGB) as applied by^37^ was used to confirm the discrimination of lithologic alterations (Fig. 6).

**Table 2:** The main band math and algebra applied to discriminate the different minerals associated with radioactive elements.

Mineral index	Band math	Spectral range	References
Sericite-illite-muscovite	(B5 + B7)/B6)]	[2145:2185] + [2235:2365][2185:2225)	^41^
Kaolinite	(B7-B5)	[2235:2365][2145:2185]	^7^
Ferrous silicate	(B5/B4))	[2145:2185][1600:1700]	^42^
Clay index	(B1-B7)/(B6-B6)	[2145:2185]·[2235:2365][2185:2225]·[2185:2225]	^42^
Argillic-non argillic	(B5/B7)		^43^
Alunite	(B4 + B6)/B5)	[1600:1700] + [2185:2225][2145:2185]	^41^
Quartz rich rocks	(B14/B12)	[8925:9275][10250:10950]	^42^
Epidote-amphibole-chlorite	(B6 + B9)/(B7 + B8)	[2185:2225] + [2360:2430][2235:2365] + [2295:2365]	^41^
Co3- epidote	(B7 + B9)/B8	[10250:10950][10950:11650]	^42^
Ferric ferrous	B3-B1	[2145:2185][760:860] + [520:600][630:690]	^41^
Alteration	(B4-B5)	[1600:1700][2145:2185]	^44^

### Mineral mapping

After the derivation of endmembers from the image and spectral signature of the USGS spectral library is validated, the entire image was mapped with minerals using a spectral angle mapper and (CEM) technique.

- **Constrained energy minimization Technique (CEM):** This technique works to find and identify the altered minerals by clarifying each mineral in a different color, such as the one whose concentration in one place indicates the severity of the change in this place and through which the place of the new exploration can be determined^15^.
- **Spectral Angle Mapper (SAM):** SAM is a method to determine the similarity between an unknown spectrum and a reference spectrum, SAM determines the resemblance between a pixel’s spectrum and each of the reference spectra. If the spectral angle is below a certain threshold (typically set as the maximum angle), the pixel is classified as representing the same kind of surface material, such as an alteration. Pixels that are more than a few radians away from the maximum angle threshold are not classified^38^.

### Arc GIS modelling

Arc GIS modelling involves integrating all output from different remote sensing and geophysical data and techniques into thematic layers. These layers were converted into raster format and assigned weights and ranks using the Analytic Hierarchy Process (AHP) statistical method. The final potential alteration anomaly zones map is created by adding criterion scores and multiplying their weight ( Table 4 in supplementary data).

### Land surface temperature (LST)

LST is influenced by the thermal distribution, emissivity in a pixel, and the wavelength range of measurement^39^. The DN (Digital Number) was converted to spectral radiances (L) using the equation by^35,40^.

1 $$Temperature= K2/(log (K1/radiance) +1)) - 273.15$$

$$Radiance \, L\lambda = ((Lmax-Lmin)/254) * (DN-1)) + Lmin) +1)$$

K1 = 774.8853, K2 = 1321.0789, Lmax = 22.00180, Lmin = 0.10033 (for Landsat 8).

### Airborne gamma-ray spectrometry (AGRS)

The AGRS survey was conducted by^45^. The survey consisted of systematically parallel flight lines oriented in the NE-SW direction, with a line spacing of one kilometer and a ground clearance of 120 meters^45^. The collected data were corrected for background radiation, changes in aircraft altitude relative to the ground, and Compton scattered gamma-rays in the potassium and uranium energy windows. These corrections were made to provide an estimate of the apparent surface concentrations of potassium (K), equivalent uranium (eU), and equivalent thorium (eTh). The corrected data were arranged for each variable in a single column in a database. Constructing a set of radioelements K, eU, and eTh-colored maps, to show the surface distribution of these radioelements and the highly anomalous zones.

The processing of the radio-spectrometric data for the studied area included the following steps:

1. Construct a set of colored maps for the radioelements K, eU, and eTh to display the surface distribution of these radioelements and highlight highly anomalous zones^45^.
2. Creating a Radioelements Composite Image (RCI).
3. Separating the radio-spectrometric measurements based on the radioactivity levels, RCI and surface geology units from published Conoco geological map.
4. Computing statistical characteristics for six selected areas, including the arithmetic mean (X), standard deviation (S), range, and checking the normality of the measurement distribution for each rock unit using coefficients of variation (CV%) and normality checks (Table 3).
5. Estimating, mapping, and interpreting the surface radioactive heat production based on the airborne gamma-ray data for various rocks in the area. The results of the anomalies are listed in Table 4.

**Table 3:** Statistical analysis of the variables in the different lithologic units of the six selected locations of the different rock units.

Area number	Area name	Lithologic units (no. of measurements)	Radioelement variables	Min	Max	X	Range	S	(CV%)
(1)	WEST G. NIKEIBA, URF EL- MIHEYIB AND URF ABU- HOMR	YG 2857	K (%)	2.2	30.6	8.9	28.3	4.1	45.7
			eU (ppm)	7.4	97.6	23.3	90.2	8.8	37.7
			eTh (ppm)	58.9	110	83.8	51.9	6.2	7.4
		MS 2083	K (%)	2.6	22.2	7.4	19.6	2.9	39
			eU (ppm)	1.2	99.9	23	98.7	15.3	66
			eTh (ppm)	19.4	113.4	83.6	93.9	12.7	15.2
		WD 273	K (%)	2.6	28.4	10.9	25.8	4.8	44.3
			eU (ppm)	0.4	93.9	25.9	93.5	13.4	51.7
			eTh (ppm)	66.2	94.1	83.1	27.9	5.1	6.2
		AG 116	K (%)	12.1	31.8	23.8	19.6	5.4	22.6
			eU (ppm)	0.1	34.7	15.1	34.6	10.8	71.6
			eTh (ppm)	58.9	82.9	73.8	23.9	5.3	7.2
(2)	G. DIHMIT AND G. UMM- SAUWAN	YG 2219	K (%)	0.1	35.3	11.8	35.2	4.47	37.8
			eU (ppm)	14.7	59.3	31.4	44.5	10.6	33.7
			eTh (ppm)	52.1	105.7	67.8	53.6	4.6	6.8
		AG 1175	K (%)	3.4	41.1	19.9	37.7	8.6	43.4
			eU (ppm)	1.7	53.6	24.4	51.8	10.5	42.8
			eTh (ppm)	36.6	111	70	74	15.8	22.6
		WD 1660	K (%)	8.8	35.8	16.9	27	6.2	36.8
			eU (ppm)	4.8	56.1	22.1	51.1	7.6	34.4
			eTh (ppm)	40	110	85	70	18	21.3
(3)	G. ABU- MARWA	MV 1079	K (%)	1.2	34.3	17.9	33.1	7.9	44
			eU (ppm)	7.1	48.8	21.3	41.7	6.6	31.2
			eTh (ppm)	64	105.2	83.3	41.2	9.1	11
		YG 3682	K (%)	2.8	44.3	22.9	41.6	6	26.2
			eU (ppm)	6.6	51.5	20.78	44.9	8.9	43.1
			eTh (ppm)	65.1	106.6	81.1	41.5	7.2	8.8
		MG 197	K (%)	8.6	32.2	21.4	23.6	6	28
			eU (ppm)	12.3	41.9	37.3	29.6	4.7	12.6
			eTh (ppm)	80.8	91.2	89.4	10.3	1.63	1.8
		WD 313	K (%)	16.5	35.4	22.1	18.9	3.3	14.9
			eU (ppm)	8.8	50	28.3	41.2	9.1	32.2
			eTh (ppm)	67.6	102.5	86.3	34.9	10.3	11.8
(4)	G. HADAIYID AND G. NUSUB	YG 3103	K (%)	0.95	29.5	10.7	28.5	4.1	38.5
			eU (ppm)	7	41	21.4	33	5.4	25.6
			eTh (ppm)	47.4	152.7	88	105	19.6	22.3
		MG 420	K (%)	1.15	21.6	8	20.5	4.2	52.4
			eU (ppm)	6.2	30.2	16.5	23.9	4.98	30.3
			eTh (ppm)	65.4	108	82.4	42.6	10.8	13.1
		MS 1109	K (%)	9.3	15.5	10.3	6.2	1.1	10.4
			eU (ppm)	8.1	41.8	20	33.7	7.1	35.3
			eTh (ppm)	62.9	118.7	80.1	55.8	10.9	13.7
		Ring complex 89	K (%)	1.86	25.9	9.6	24.1	5.4	55.8
			eU (ppm)	9.8	20.8	14.6	11	3.1	21
			eTh (ppm)	66	99.1	77	33	6.9	8.9
		OG 326	K (%)	9.9	15	12.3	5.1	1.7	14.1
			eU (ppm)	15.2	40.8	24.6	25.6	5.7	23
			eTh (ppm)	69.1	80.2	75.4	11	3.4	4.4
		MV 108	K (%)	3.9	17.2	7.4	13.3	2.8	38.2
			eU (ppm)	8.5	22.4	14.9	13.8	4.5	30.6
			eTh (ppm)	66.2	107.6	83.1	41.4	13.1	15.7
(5)	G. ABU RAHANY, G. BAIAT AND G. RAIATIT	Meta 3694	K (%)	0.1	31.7	10.6	31.6	6.6	62.7
			eU (ppm)	7.7	21.6	13.1	13.8	3.8	29
			eTh (ppm)	85.9	101.6	93.5	15.7	4.6	4.9
		YG 418	K (%)	0.1	29.9	9.7	30.2	5.9	61.5
			eU (ppm)	8.4	20.9	13.3	12.5	3.5	26.3
			eTh (ppm)	88.7	98.6	90.1	9.8	0.98	1.1
		OG 528	K (%)	2.6	24.2	8	21.6	4.2	52.4
			eU (ppm)	9.3	21.8	13.7	12.4	4.2	30.5
			eTh (ppm)	87.6	100.7	93.5	13.1	5	5.3
		WD 866	K (%)	2.8	27.1	11.8	24.3	4.3	36.3
			eU (ppm)	8.2	21.3	12.8	13.2	2.9	22.9
			eTh (ppm)	87.7	101.3	13.6	95.9	3.6	3.7
(6)	G. H0MRAT EL FIG	YG 2415	K (%)	0.2	32.5	17	32.3	6.8	40.2
			eU (ppm)	3.5	38.8	20.9	35.3	4.6	22
			eTh (ppm)	54.4	254	89	200	22.5	25.3
		Meta 755	K (%)	14.6	21.2	19.8	6.6	0.9	4.6
			eU (ppm)	10.3	21.9	18.4	11.6	2.3	12.6
			eTh (ppm)	55.6	90.5	72.1	34.9	8	11
		WD 2177	K (%)	10.5	35.4	22.8	24.8	3.3	14.5
			eU (ppm)	2.9	49.8	20.8	46.9	8.4	40.8
			eTh (ppm)	52.9	186.4	83.1	133.4	21.7	26.1

Explanation: Min. is minimum of variables, Max. is maximum of variables, YG is younger granites, Meta is metamorphic rocks, OG is older granites, Ring is ring complex, MG is Metagabbro, MV is metavolcanic, MS is metasediments, AG is Abu Aggag Formation, WD is wadi deposits, X is Arithmetic mean, S is standard deviation and C.V. is Variability Coefficient.

**Table 4:** Average density and estimated radioactive heat production corresponding to each rock unit.

A. No	Area name	X1min Y1min	X2max Y2max	LU	Sample NO	Average density (g/cm^3^)	Calculated radioactive heat production (µWm^-3^)					
							Min	Max	X	S	CV%	Estimated general level
(1)	WEST G. NIKEIBA, URF EL- MIHEYIB AND URF ABU- HOMR	6,417,697 2,634,277	640,876 2,651,885	YG	2857	2.64	0.7	3.1	1.3	0.2	18	High level
				MS	2083	2.62	0.2	3.2	1.2	0.4	33.9	High level
				WD	373	1.92	0.5	2.2	0.9	0.2	25	Intermediate level
				AG	116	2.41	0.7	1.4	1	0.2	20	Intermediate level
(2)	G. DIHMIT AND G. UMM- SAUWAN	514,964 2,617,305	538,097 2,634,868	YG	2219	2.64	0.9	1.9	1.3	0.2	18	High level
				AG	1175	2.41	0.7	1.6	1.1	0.17	15	Intermediate level
				WD	1660	1.92	0.6	1.3	0.9	0.08	9	Intermediate level
(3)	G. ABU- MARWA	523,991 2,545,994	547,133 2,563,556	MV	1079	2.7	0.7	1.9	1.1	0.22	17	Intermediate level
				YG	3682	2.64	0.7	1.9	1.3	0.24	18	High level
				MG	197	2.93	0.8	1.4	1	0.09	9	Intermediate level
				WD	313	1.92	0.6	1.4	1.09	0.13	12	Intermediate level
(4)	G. HADAIYID AND G. NUSUB	553,260 2,546,104	576,238 2,563,556	YG	3105	2.64	0.7	1.8	1.23	0.22	17	High level
				MG	109	2.93	0.8	1.5	1	0.2	20	Intermediate level
				MS	1109	2.62	0.7	1.6	1.1	0.18	16	Intermediate level
				Ring	90	2.76	0.8	1.3	1	0.11	11	Intermediate level
				OG	327	2.64	0.96	1.6	1.25	0.14	11	High level
				MV	420	2.7	0.7	1.5	1	0.19	18	Intermediate level
(5)	G. ABU RAHANY, G. BAIAT AND G. RAIATIT	594,073 2,545,994	617,105 2,563,611	Meta	3695	2.72	0.8	1.3	1.1	0.13	12	Intermediate level
				YG	419	2.64	0.8	1.2	1	0.1	10	Intermediate level
				OG	519	2.64	0.8	1.2	1.05	0.08	7.7	Intermediate level
				WD	867	1.92	0.6	0.9	0.7	0.07	9	Low level
(6)	G. H0MRAT EL FIG	624,874 2,546,104	647,848 2,563,678	YG	755	2.64	0.8	2.5	1.2	0.2	16	High level
				Meta	2415	2.72	0.9	1.3	1.1	1	8	Intermediate level
				WD	2177	1.92	0.6	1.6	0.94	0.2	24	Intermediate level

Explanation: A. No. is area Number, X1 min. is X minimum of southwestern corner, Y1 min. is Y minimum of southwestern corner, X2 max. is X maximum of northeastern corner, Y2 max. is Y maximum of northeastern corner, YG is younger granites, Meta is metamorphic rocks, OG is older granites, Ring is ring complex, MG is Metagabbro, MV is metavolcanic, MS is metasediments, AG is Abu Aggag Formation, WD is wadi deposits. Min. is the Minimum value, Max. is the Maximum value, X is the Arithmetic mean, S is the standard deviation, C.V. is the Coefficient of Variability, the Low level (values reaching less than 0.8 mWm^-3^), Intermediate level (values ranging from 0.8 to 1.1 mWm^-3^) and High level (values attaining more than 1.2 mWm^-3^).

## Results and discussion

### Remote sensing

#### Alteration zone detection and mineral discrimination

In this study, remote sensing technology has been combined with spectroscopy to investigate the electromagnetic spectrum in the range of 2-14 um. The goal is to discriminate and detect minerals, which are the main components of rocks, and to monitor the concentration of radioactive elements associated with heat generation. To detect alteration zones and discriminate minerals, Principal Component Analysis (PCA) was applied to reduce spectral discrepancies while preserving important information. The resulting eigenvalues and eigenvectors were analyzed to identify minerals based on their positive or negative signals. By studying the lightness or darkness of specific bands, it is possible to determine which Principal Components (PCs) could be used to extract the desired targets. For example, within PC4, there are two opposing signed loadings in B7 (-0.163719), B2 (-0.272846), and B6 (0.934732). A negative sign (high reflectance) in B7 and a positive sign (high absorption) in B6 indicate that illite and muscovite will appear as dark pixels in the PC4 image (Table 3 in supplementary data).

Band ratio spectral enhancement approaches were utilized to improve spectral contrast and successfully map altered zones. Various band ratios were applied to multispectral data, resulting in the discrimination of minerals such as kaolinite, Fe-OH (chlorite), Mg-OH (epidote, amphiboles), calcite, dolomite, and alunite. The ASTER thermal bands (TIR) proved effective in separating silicate minerals like quartz (Qz), which is often associated with radioactive mineralization. Sheet silicate groups (kaolinite, muscovite, clay, and mica minerals) have a prominent absorption peak at 1.4 and 2.3 µm. Bands 7 and 6 with Band ratio (B1-B7)/(B6-B6) show good results to discriminate the potassium-rich clay minerals which appeared as bright color (Fig. 4). Making a density slice of the clay bands algebra for determining the concentration of clay minerals showed the concentration of clay minerals in the places of mineral changes and alteration that correspond to the concentration of radioactive materials. The CO_3_- epidote has a prominent absorption peak at 2.3 µm with ((B7+B9)/B8) ratio appear as dark patches can discriminate the rock with high abundance of CO_3_-epidote but (B6+B9)/(B7+B8) ratio does not give a good result for epidote-amphibole-chlorite discrimination (Fig. 4). The Fe-oxides (hematite, magnetite) absorption bands near 0.9 in bands 2 and 5, ratio (B3-B1) show the Ferric ferrous but the ratio (B5/B3) + (B1/B2) cannot give results to detect Ferric-ferrous oxides. Quartz (Qz) spectral signature is high in TIR spectral range (B13 and B14), but with no significant signature in the VNIR-SWIR range, Table 2. Illite spectral signature is high in VNIR-SWIR spectral range (B1 and B7) and low in (B6 and B8) but with no significant signature in TIR spectral range. ((B5+B7)/B6) ratio signifies rock abundant in Sericite-illite-muscovite (Fig. 4).

Band math calculations, such as (B7xB4/B6xB6) showed loss of K and addition of Hydroxyl (OH) in the alteration zones. These changes were formed by temperature variation and chemical alteration. Additionally, specific band ratios, such as (B1-B7)/(B6-B6), were used to discriminate clay mineral-rich areas enriched with potassium (Fig. 5).

**Fig. 7:**
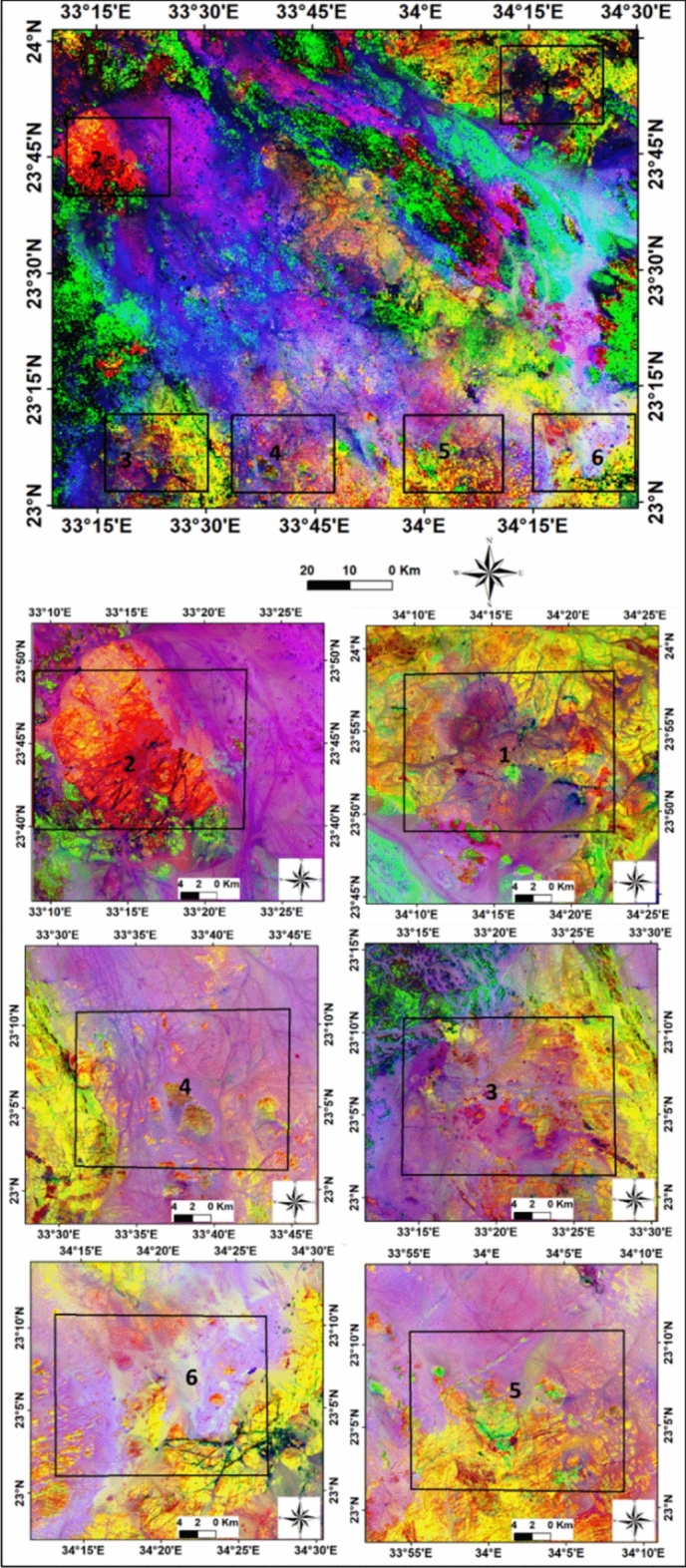
Aster 1 level band ratios image (b6/b4, b7/b3, b3/b1) in RGB and enlarged selected parts of Gabal Abu Hashim area and its surroundings, SED, Egypt.

By assigning appropriate colors, specific lithologies could be rendered in the images. The results indicated higher concentrations of illite, chlorite, kaolinite, alunite, and sericite minerals, while iron minerals were less abundant. Different image processing techniques, including various color composites, such as landsat ratios (B5/B7, B3/B5, B3/B1) delineate altered minerals which appear as purple with maroon color, (Fig. 6). The false-color composite ASTER band ratios (B6/B4, B7/B3, B3/B1),were employed to distinguish altered zones,. The alteration zones in the selected six areas, from 1 to 6, were emphasized with reddish-yellow color, (Fig. 7). The interpretation of processed satellite data was validated using the integration of geological map of the study area, field investigations and ground truth information.

This helped assess the spatial distribution of different lithological units and validate the findings of the remote sensing analysis (Tables 1 and 2 in supplementary data).

**Fig. 8:**
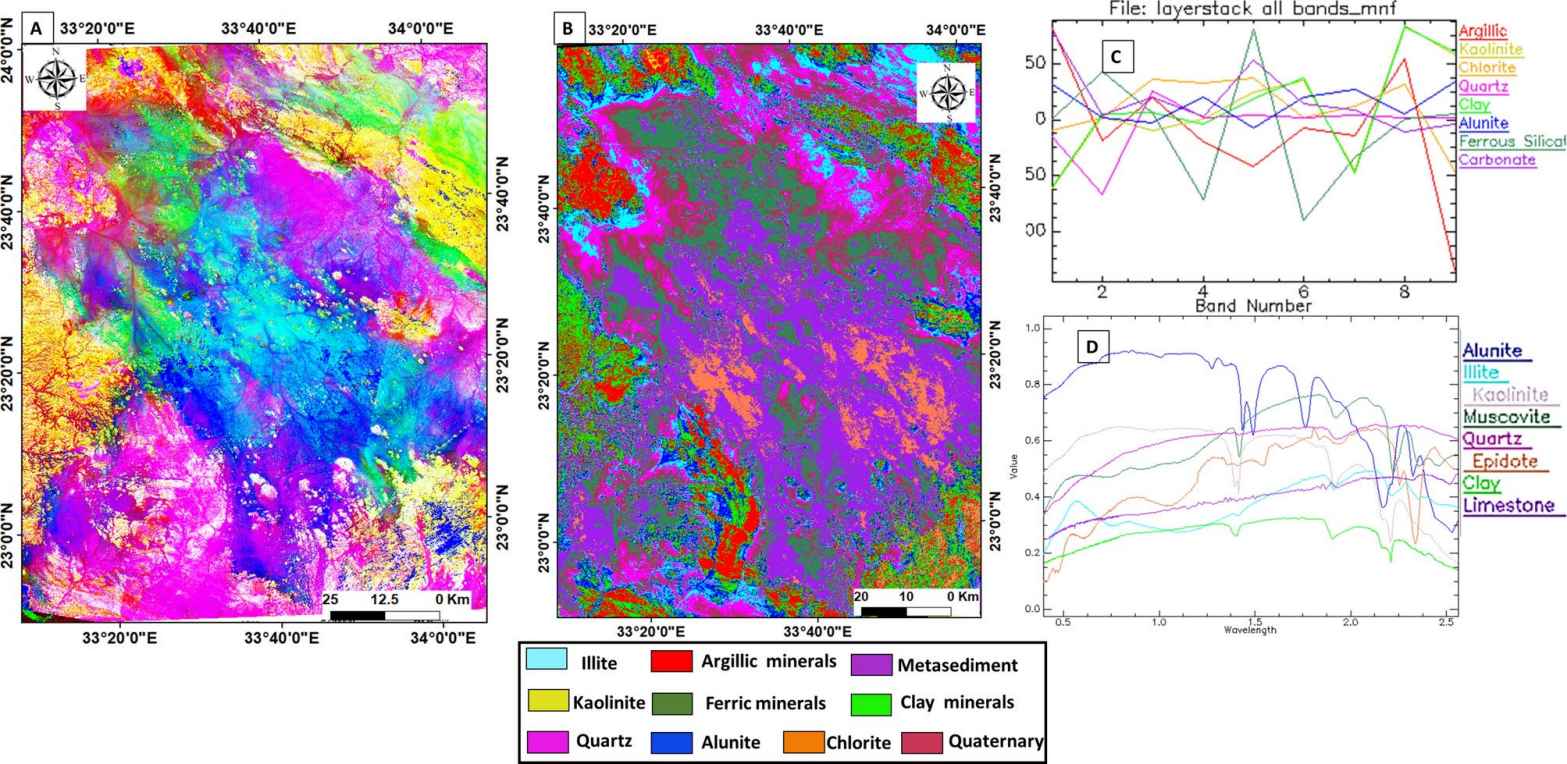
(**A**) The false-color image of MNF 7,5,3 RGB, and (**B**) the SAM classification of the identified end-members of interest for mineral mapping throughout the whole image, and **C** and **D** The spectral signature curve and reflection response of various minerals were found to indicate geothermal alteration.

The Spectral Angle Mapper (SAM) technique was utilized to separate end members with known spectral fingerprints from unknown members. By matching endmember spectra with those in the USGS database, classification can be performed to map alteration minerals associated with uranium occurrences, such as kaolinite, illite, hematite, and chlorite (Fig. 8).

**Fig. 9:**
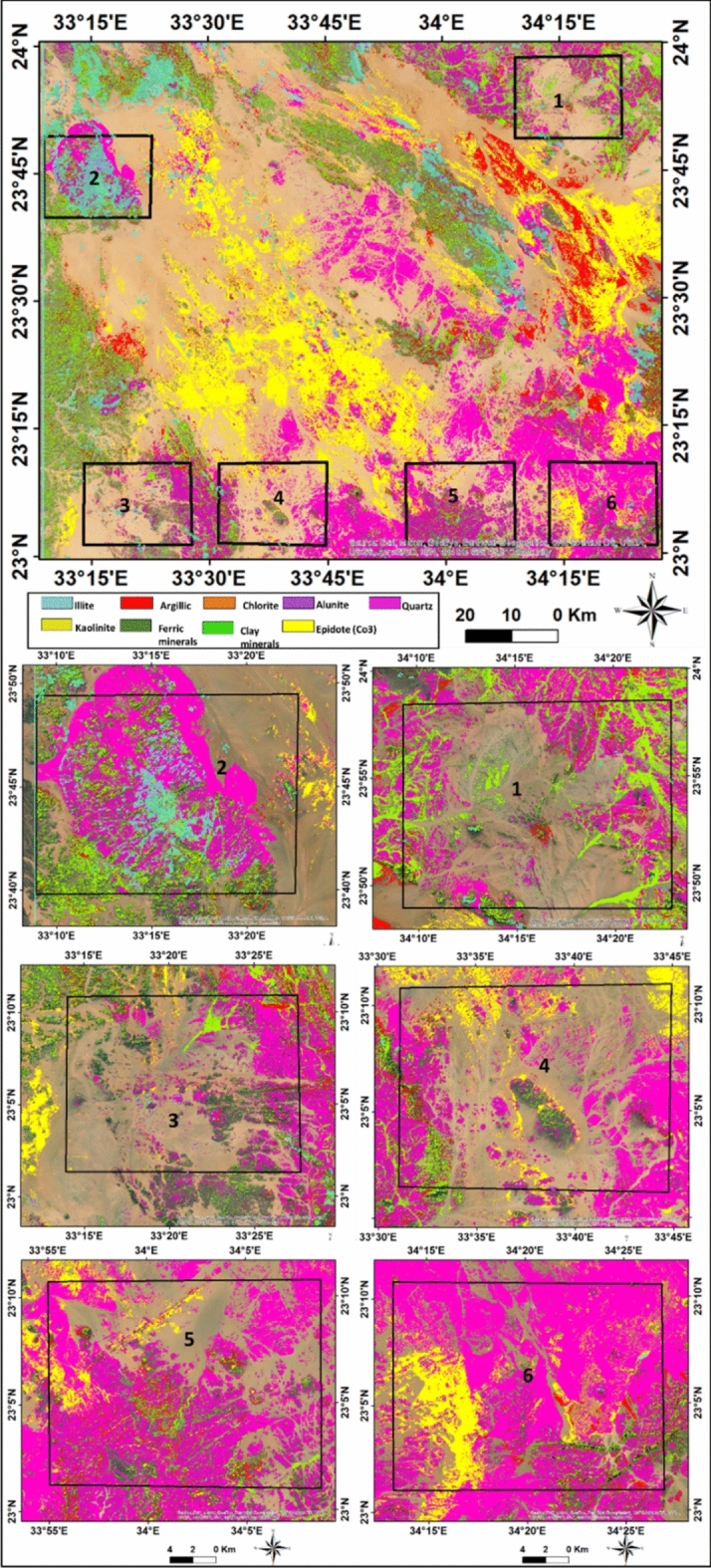
The final result of alteration anomaly minerals slices from the different processes overlaid on the true-color satellite image of the Gabal Abu Hashim area and its surrounding parts, SED, Egypt.

The analysis of multiple mineral interference indicates the extent of rock alteration and the concentration of radioactive elements in the study area. In the color-coded map (Fig. 9), different minerals are represented by specific colors. Alunite is shown as purple, clay as green, quartz (Qz) as pink, epidote as yellow, argillic ferric minerals as dark green, illite as cyan, and kaolinite as pale yellow.

**Fig. 10:**
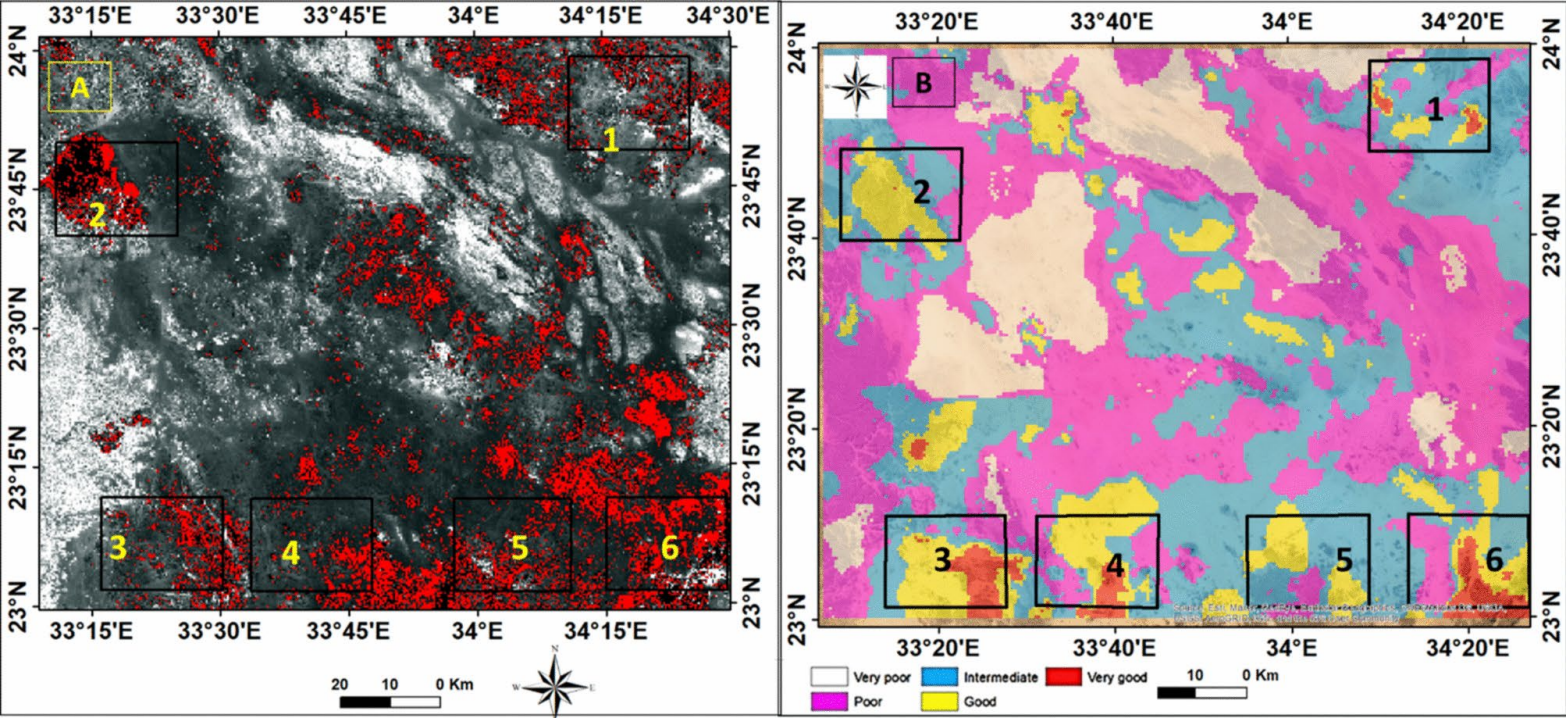
(**A**) Resulted alteration anomaly slice overlaid on the satellite image of the study area, (**B**) Radioelement potentiality zoning map of Gabal Abu Hashim and its surrounding areas, SED, Egypt.

The areas with highest rocks alteration, which correspond to the occurrence of radioactive minerals, are highlighted in red to distinguish them from fresh rocks and non-potential regions (Fig. 10a).

The study area exhibits six relatively high uranium-bearing zones. The maps depict the concentrations of radioactive substances in various rocks and divide the area into five zones: very poor, poor, medium, good, and high concentrations (Fig. 10b). The concentration levels vary based on the rock types. high concentrations are found in younger granites, medium concentrations are found in older granites, and low concentrations are observed in tertiary metavolcanic rocks.

The radiation levels in the tertiary metavolcanic rocks remain within safe limits for human exposure and are lower than the maximum permissible levels from natural gamma radiation sources. The resulting maps provide valuable information about the distribution of radioactive elements within the altered rocks.

#### GIS model result

The interpolation of measured data points was used to define the spatial variability over the area domain. The IDW technique was used to interpolate and map the radioelements abundance which used as thematic inputto weighted overlay model with other controlling factors, the model was calibrated with measured points in the field. IDW of field radio spectrometer spatial concentration mapping and remote sensing data were integrated with a GIS framework to classify alteration prospects into five potential zones namely;, very good, good, intermediate, poor, and very poor. The zone of very good anomaly and alteration appears in the limbs of the study area at northwestern, southwestern, northeastern parts. The radioactive elements have great environmental implications, and their increase is considered harmful to the environment, (Fig. 10).

#### Structural interpretation

Structure geology is the first stage of any regional survey for new mineral exploration. Incorporating tectonic reworking and geological processes related to radioactive mineralization can be used to explore alterations and their relation to radioactive minerals occurrence. . The shear zone hosted radioactive mineral deposits associated with silicate veins and K- rich clay minerals are stained with different colors based on the types of alteration products and anomalies. Alteration-filling fractures and shear zones are related to these anomalies. Specific alteration phenomena like silicification, hematitization, and kaolinization are shown in the mineralized shear zones.

**Fig. 11:**
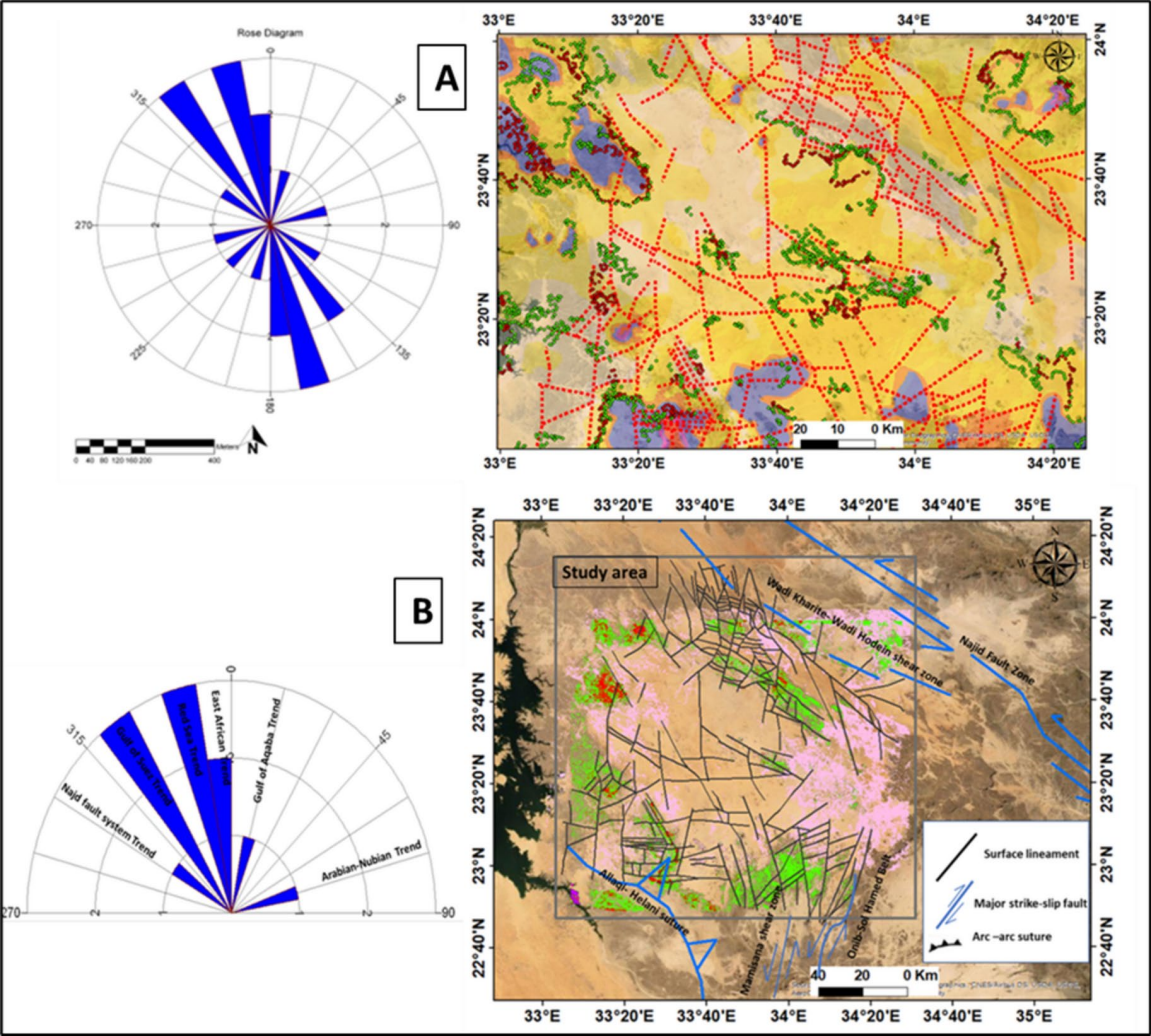
(**A**) The extracted lineaments dropped over alteration with inset azimuth-frequency rose diagram, (**B**) the result of alteration mineral slices overlayed on satellite image with surface lineament and major regional faults, and azimuth diagram shows the main tectonic trends of Gabal Abu Hashim area and its surrounding parts, SED, Egypt.

The study area exhibits four main fault trends, namely NW, NE, N-S, and ENE, (Fig. 11A). These structural lineaments play a crucial role in the deformation history along the Red Sea margin, particularly in the Nubian side. Additionally, minor trends in the NNW-SSE and NNE-SSW directions are also present.

The NW-SE trend is identified as the primary lineament trend, which corresponds to the sela shear stress, contolling the structural framework of the study area and the Nubian side in general. This trend aligns with the Najid Fault Zone, known for its left-lateral strike-slip movement, and the Wadi Kharite-Wadi Hodein shear zone. The NNE-SSW structural trend is parallel to the regional movement caused by the Hamisana shear zone and Onib-Sol Hamed Belt, which controls the alteration zone in the sites 1 to 6 (Fig. 11B).

#### Lithological units’ detection

For the detection of lithological units, multispectral satellite images were employed to map the geological and lithological features, mineral alterations, and geological structures in the study area. Various remote sensing techniques were applied, including mono-spectral analysis of ASTER images and the methods mentioned earlier in the flowchart. These techniques aim to differentiate between variable rocks, identify lithological alterations associated with the presence of radioactive elements, and study their concentrations and distribution in relation to the geological structures and tectonic evolution of the region.

The RGB combination of Principal Component Images (PCI) PC1, PC2, and PC3 were utilized to enhance the image and discriminate lithologies. Principal Component Analysis (PCA) was applied to rearrange the bands based on increasing variance. Endmembers were identified using the USGS spectral library, and their spectral analysis scores were matched with known and unknown spectra. These endmembers were then used to construct a mineral abundance map using the Spectral Angle Mapper (SAM) approach. The 1:50,000 scale Geological Map was employed to validate the final maps.

GIS technology, specifically overlay-weighted criteria, was utilized to create zonation for radioactive concentrations and potentiality. The results obtained from satellite image analysis were overlaid and linked with field results and radiometric measurements (U, Th, and K) to evaluate radiation levels in the region. The results from these processes align with field measurements, accurately delineating the locations of radioactive elements using remote sensing data and images (Fig. 10).

Indicator minerals of the alteration zone, such as chlorite, alunite, illite, kaolinite, and sericite, are common in the study area, while ferrous minerals and epidote are less abundant. The Abu Aggag Formation coincides with the alteration and metamorphism is observed in the southern part of the study area, along with the ring complex and younger granite.

The topography, structures, regional tectonic movements, and other geological factors influenced the efficiency of mineral alteration. The alteration zones with high radiance values were detected by modeling these factors and linking the results, map analyses, and statistics obtained from field experiments and remote sensing techniques The model output suggests that the northern and southeastern regions are highly efficient in terms of alteration, which corresponds to areas with high fault density especially that affected by the shear zone. (Fig. 11). The NNE-SSW structural trend is parallel to the regional movement caused by the Hamisana shear zone and Onib-Sol Hamed Belt, which controls the alteration zone in sites 1 to 6.The NW-SE and NNE-SSW trends play a significant role in controlling the distribution of radioelements and radioactive minerals in a complex setting.

The LST was estimated based on Equation (1) from the Landsat 8 image to estimate the radiance and earth heat production values for each rock unit. It reaches up to 56 Celsius at the selected six areas.

### Airborne gamma-ray spectrometry (AGRS)

#### Qualitative interpretation of the AGRS maps

The qualitative interpretation of the AGRS (Airborne Gamma Ray Spectrometry) maps provides valuable insights into the distribution of radioelements (K, eTh, and eU) in the study area. The maps reveal the spatial variations of these elements across different rock units and anomalous zones. The values of K, eTh, and eU are multiplied by 10 for better visualization. Table 3.

##### **a. Radioelements maps**

**Fig. 12:**
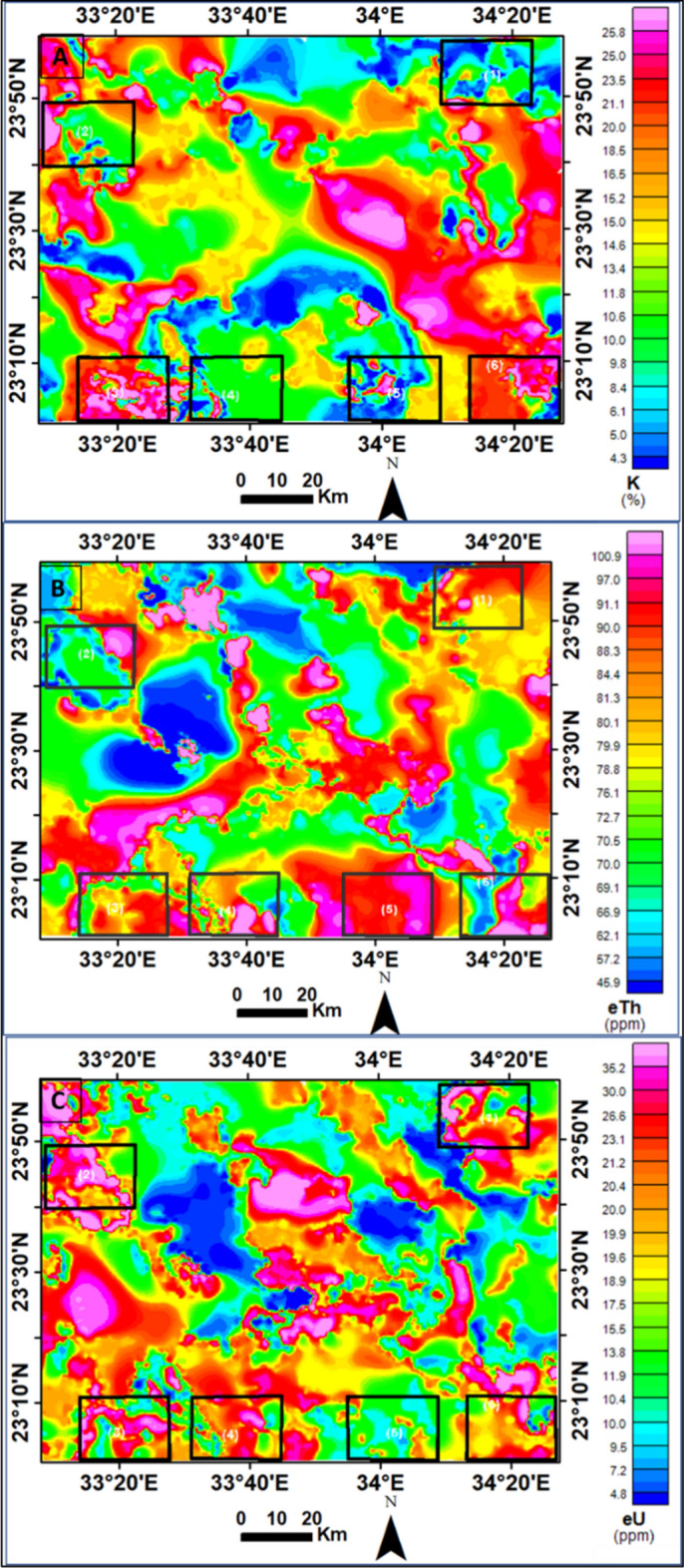
(**A**) Airborne colored map of K in % (**B**) Airborne colored map of eTh in ppm (**C**) Airborne colored map of eU in ppm of Gabal Abu Hashim area and its surrounding parts, SED, Egypt,^45^.

The K map (Fig. 12A) illustrates the relative potassium concentrations in the area. The wadi sediments, Abu Aggag Formation, Um Barmil Formation, and metavolcanic exhibit the lowest potassium contents (less than 10%). Intermediate potassium levels ranging from 10 to 20% are associated with metagabbro, metasediments, and the Timsah Formation. The highest potassium levels (more than 20%) ??? are found in the younger granites, ring complexes, metamorphic rocks, and older granites, which can be delineated.

The eTh map (Fig. 12B) demonstrates the distribution of thorium concentrations. The younger granites, older granites, and ring complexes display higher eTh contents. The map is divided into three levels: the highest level, ranging up to 90 ppm, is associated with the aforementioned rocks and metamorphic rocks. The intermediate level (70 to 90 ppm) is limited to wadi sediments, the Abu Aggag Formation, and the Timsah Formation. The lowest eTh values (less than 70 ppm) are primarily related to the Um Barmil Formation, metagabbro, metasediments, Tertiary basalt, and metavolcanic.

Similarly, the eU map (Fig. 12C) is divided into three levels. The higher level, ranging up to 20 ppm, is associated with the younger granites, older granites, ring complex, and metamorphic rocks. The intermediate level (10 to 20 ppm) is mainly found in the Timsah Formation and Wadi sediments. The Abu Aggag Formation, Um Barmil Formation, metagabbro, metasediments, Tertiary basalt, and metavolcanic have lower eU levels, with values less than 10 ppm.

##### **b. Ternary map**

**Fig. 13:**
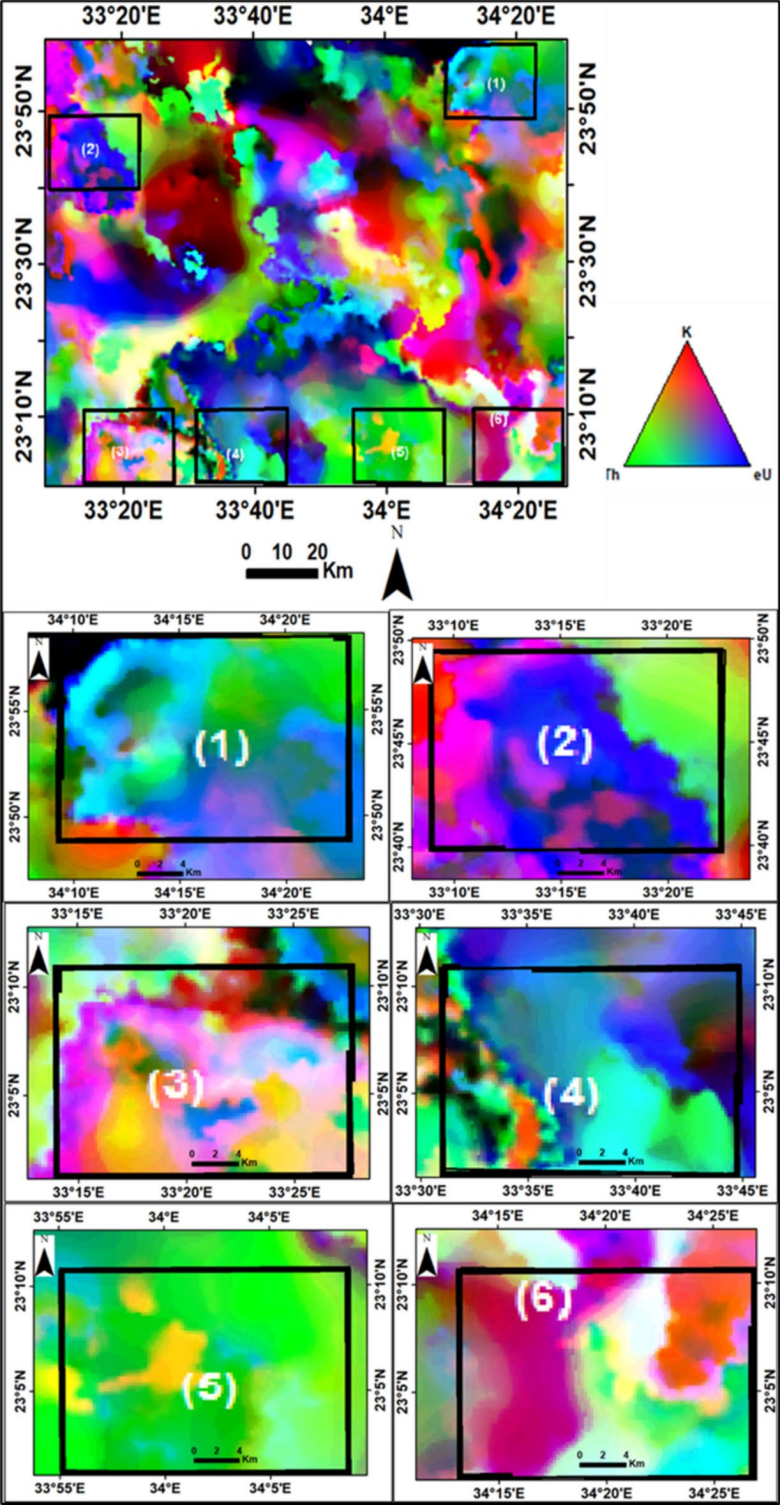
Radioelements composite image (RCI) or Ternary map and their selected parts (1, 2, 3, 4, 5, and 6) of Gabal Abu Hashim area and its surrounding parts, SED, Egypt.

A ternary map, the radioelements composite image (RCI), was generated by modulating the colors of the K, eTh, and eU grids. This map helps in separating and delineating different geological units and identifying enriched or poor zones of the three radioelements. The RCI (Fig. 13) exhibits three distinct characteristics^10,46^. The bright colors represent rocks rich in all three elements (K, eU, and eTh). The triangular legend indicates a 100% concentration of each radioelement, and the colors within the triangle reflect specific radioelement ratios.

The younger granites, older granites, ring complex, and metamorphic rocks exhibit strong radio-spectrometric responses and distinctive elemental differences, which are visible on the ternary radioelements map. These rocks can be easily distinguished from surrounding units and are spatially correlated with zones of anomalously high K, eU, and eTh levels (the white color parts on the map). Additionally, specific areas (parts No. 1, 2, and 4) are enriched with eU (blue color), while parts No. 1, 2, and 5 are enriched with eTh (green color). Black areas on the ternary map indicate weaker radioelement contents, representing low radioactive rocks such as metasediments, Um Barmil Formation, Tertiary basalt, Abu Aggag Formation, and metavolcanic.

### Quantitative interpretation of the AGRS data

Table 3 presents the results of the standard statistics applied to the spectrometric data. The statistics include means, minimums, maximums, standard deviations, and normality checks (CV%) for each variable. The coefficient of variation (CV%) is calculated as the ratio of the standard deviation to the mean, expressed as a percentage^47^ . Statistical computation was applied to the original spectrometric data, without applying any transformation, according to the recommendation given by^48^.

**Fig. 14:**
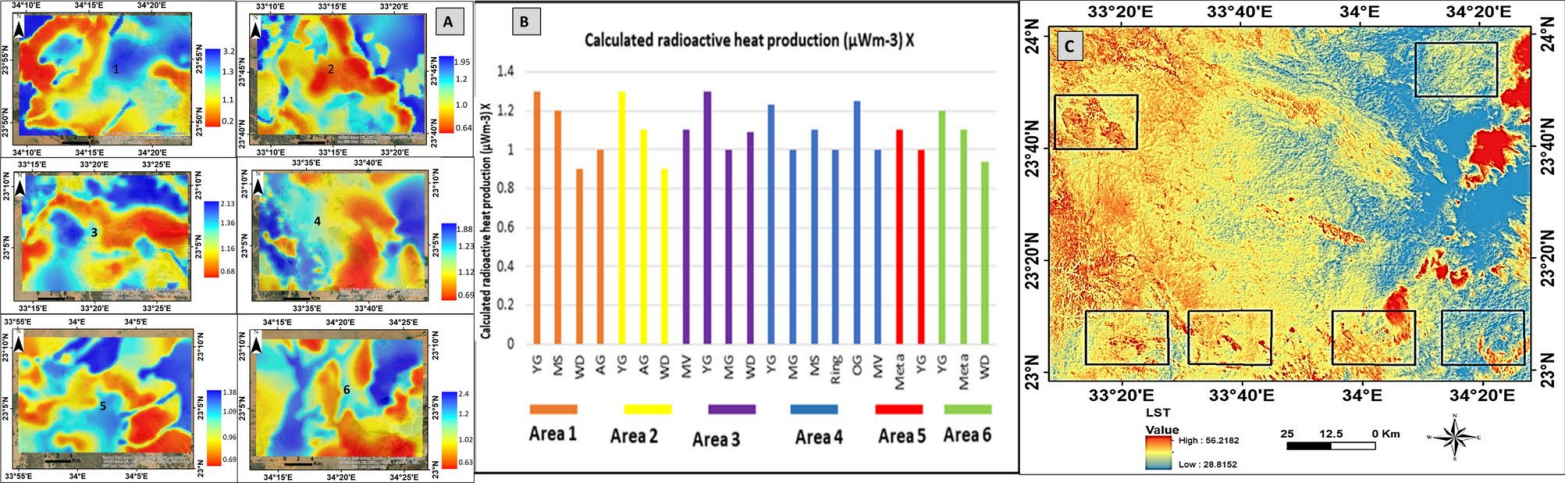
(**A**) Calculated radioactive heat production color map of Gabal Abu Hashim area and its surrounding parts, SED, Egypt, (**B**) Graphical chart of calculated radioactive heat production and related rock type. (**C**) Calculated land surface temperature (LST) and surface thermal radiance calculated from Landsat 8.

To estimate the radiogenic heat production (RHP) for different rock units in the selected parts, the means, minimums, maximums, standard deviations, and normality checks for each variable were computed. The RHP provides a measure of the rock’s radioelement concentration relative to the background levels.

(Table. 4), and (Fig. 14), illustrates the RHP values for each rock unit, based on the computed statistics. The RHP values indicate the deviation of the radioelements concentrations from the background levels. High RHP values suggest elevated radioelement concentrations, while low values indicate low concentrations.

In determining the normal distribution of the variables, the coefficient of variation (CV%) was used. If the CV% is less than 100% for a certain variable value in the study area, the variable tends to exhibit a normal distribution, according to the following equation:

2 $$CV \% = (\sigma /X) \times 100$$

where: σ is the standard deviation and X is the arithmetic mean.

The quantitative calculations in the study focused on assessing the homogeneity of the distribution of spectrometric variables among different lithologic rock units. Lower values of the coefficient of variation (CV%) indicate a higher degree of homogeneity in the distribution of the variables. In this study, the CV% values for eU are relatively higher compared to those for eTh and K in almost all rock units, indicating a lower degree of homogeneity in the distribution of thorium compared to the other variables. On the other hand, the lowest CV% values for eU, eTh, and K indicate a high degree of homogeneity in their distribution.

The statistical treatment of the calculated (RHP) from gamma-ray spectrometric data mainly relies on the application of the coefficient of variation (CV%) technique. The CV% helps assess the variability and homogeneity of the radioelement concentrations within the rock units.

To estimate and map the radioactive heat production (A), an empirical relationship published by^49^ is used. The expression relates the radioactive heat production to e??? concentrations of eU, eTh, and K in the rock sample, as well as the dry density of the rock. The formula is as follows:

3 $$\text{A}\left({\mu} \text{W}{\text{m}}^{-3}\right)=\rho \left(0.0952{\text{C}}_{\text{U}}+0.0256{\text{C}}_{\text{Th}}+0.0348{\text{C}}_{\text{K}}\right),$$

where: ρ is the density of dry rock (g/cm3) and C is concentration, C (U, Th, and K) are the concentrations of eU and eTh in ppm and K in %, respectively.

The radioactive heat production can be calculated using laboratory measurements of radioelement concentrations^50^ or directly from gamma-ray logs^51^. Additionally, it can be estimated from airborne gamma-ray data. However, before calculating the radioactive heat production from aerial gamma-ray data, it is crucial to carefully define the rock type and its limits and determine the average density of the rock^20,52–54^. 

The current research inferred the density for each rock unit roughly (Table 4) using the densities supplied by^55^ and various online sites in this study. It was quite difficult to calculate the statistical average density value for each rock unit. Radioactive heat production values for each rock unit were estimated based on Eq. (3). The results are summarized in Table 4 and illustrated as a map of the radioactive heat production for the selected six parts in (Fig. 14). The radioactive heat production varies, from 0.2 µWm^-3^ to 3.2 µWm^-3^ in the study area. The higher average values (Table 4) are obtained for younger granitic and older granitic rocks (up to 1.3 µWm^-3^), whereas the lowest average value is obtained for wadi sediments (less than 0.9 µWm^-3^) except the wadi deposits of part 3, which has RHP values of more than 1 µWm^-3^.The integration of geophysical data and remote sensing information enhances the accuracy of geothermal resource assessments. By measuring radioelement concentrations and mapping thermal alteration minerals, potential geothermal energy zones can be efficiently identified . This approach not only supports the exploration of geothermal resources but also contributes to a better understanding of the Earth’s internal heat dynamics, ultimately aiding in the transition to sustainable energy sources.

## Conclusions

The study successfully demonstrates the effectiveness of combining remote sensing technology with spectroscopy for mineral discrimination and alteration zone detection. The application of Principal Component Analysis (PCA) and band ratio spectral enhancement approaches are valuable in identifying and mapping various minerals and alteration zones. Principal Component Analysis (PCA) effectively reduced spectral discrepancies while preserving critical information, enabling the identification of minerals based on their spectral signatures, such as illite and muscovite. Various band ratios were applied to multispectral data, enhancing spectral contrast and enabling the discrimination of minerals like kaolinite, Fe-OH, Mg-OH, calcite, dolomite, and alunite. The ASTER thermal bands (TIR) were particularly effective in separating silicate minerals like quartz. The study found common alteration minerals indicators like chlorite, alunite, illite, kaolinite, and sericite in the alteration zone, while ferrous minerals and epidote are less abundant. Band math calculations and specific band ratios revealed that the alteration zones are influenced by temperature variations. These zones are associated with high concentrations of radioactive elements. The structural geology analysis identified the main fault trends: NW–SE, NNE-SSW, and ENE, which are crucial for understanding the deformation history in the area. These trends control the distribution of radioelements and probably other valuable minerals in the study area.

AGRS maps offer spatial distribution of radioelements across rock units and anomalous zones. The study revealed that wadi sediments, Abu Aggag Formation, Um Barmil Formation, and metavolcanic rocks have the lowest potassium content, while younger granites, ring complexes, metamorphic rocks, and older granites have the highest thorium concentrations. The radioelements composite image (RCI) helps identify the zones of high or low K, eTh, and eU contents in the rock units. It distinguishes between younger, older, ring complexes, and metamorphic rocks, providing valuable insights for mineral exploration and environmental assessments.. High RHP values indicated elevated radioelement concentrations, while low values implied lower concentrations. There is a great congruence between the locations and distributions of radioactive elements from gamma-ray spectrometry measurements and the results of distribution and potentiality of alteration zones from remote sensing applications. The GIS model effectively used Inverse Distance Weighting to map the spatial variability of radioelements in a study area. It classified alteration prospects into five zones, with zones with good anomalies in northwestern, southwestern, and northeastern regions. Landsat 8 images estimated Land Surface Temperature (LST) values, reaching up to 56 °C in areas with high radioactivity and alteration, especially in northern and southeastern regions of high fault density.

Generally, younger granite, ring complex, and metamorphic rocks have the highest quantities of radioactivity in the study area. The study of selected six areas showed that there is a close relationship between the RHP distribution of radioactive elements, structures, and alteration zones. The high RHP parts , the LST and earth emission calculated from thermal remote sensing mapping are associated with the younger granite, older granite, and metamorphic rocks.

## Electronic supplementary material

Below is the link to the electronic supplementary material.

Supplementary Information.

## Data Availability

This article has no associated data, and all the data used in this study are present in the article.
