# Supplementary material for: Exploring geothermal energy prospects through radioelement enrichment zones in Gabal Abu Hashim area in southeastern Aswan by geophysical and remote sensing data integration
Source: Sci Rep. 2024 Nov 7;14:27043. doi: 10.1038/s41598-024-77393-8 (PMC11543932; doi:10.1038/s41598-024-77393-8)
Supplement: Supplementary file 1 — Supplementary Information. [file 41598_2024_77393_MOESM1_ESM.docx]

**Table1: the details of the status and behavior of different bands' reflectance response to different characteristic minerals associated with radioactive elements.**

| **minerals** | **VNIR-SWIR (reflectance)** | |  | **TIR (Emittance)** | |
| --- | --- | --- | --- | --- | --- |
|  | **High** | **Low** |  | **High** | **Low** |
| Kaolinite | Band 3 | Band 6 |  |  |  |
|  | Band 7 | Band 9 |  |  |  |
| Chlorite | Band 1 | Band 2 |  |  |  |
|  | Band5 | Band 8 |  |  |  |
| Illite | Band 1 | Band 6 |  |  |  |
|  | Band 7 | Band 8 |  |  |  |
| Ferrous | Band5 | Band 3 |  |  |  |
|  | Band 8 | Band 6 |  |  |  |
| Quartz | no spectral features character | |  | Band 10 | Band 11 |
|  |  |  |  | Band 12 | Band 14 |

**Table 2: the main statistics of different characteristic minerals associated with radioactive elements.**

| **Basic Stats** | **Min** | **Max** | **Mean** | **StdDev** |
| --- | --- | --- | --- | --- |
| **Clay** | **0.579** | **1.3202** | **0.5955** | **0.036** |
| **0Allunite** | **-1918** | **2012** | **1.6486** | **1.4008** |
| **Illite-Muscovite** | **-25692** | **26192** | **2.1988** | **2.1432** |
| **Ferrous** | **-10.4431** | **7.0902** | **0.527** | **0.1323** |
| **Carbonate** | **-110** | **107** | **3.9757** | **3.2389** |

**Table 3: The main Eigenvector and eigenvalues of PCA of ASTER data.**

| Eigenvector Band 1 Band 2 Band 3 Band 4 Band 5 Band 6 Band 7 Band 8 Band 9 |
| --- |
| PCA1 0.060959 0.068330 0.062691 0.034621 0.045048 0.066664 0.278297 -0.950033 0.000551 |
| PCA 2 -0.132410 -0.192783 -0.179237 -0.103768 -0.119691 -0.175594 -0.871941 -0.311390 -0.002364 |
| PCA 3 0.463617 0.594447 0.515486 0.041537 0.099658 0.149892 -0.362694 0.017032 0.002212 |
| PCA 4 0.230031 0.150376 0.075417 -0.272846 -0.430212 -0.793949 0.169806 -0.005764 -0.006007 |
| PCA 5 -0.796152 0.167771 0.567740 -0.050241 -0.035481 -0.107682 0.003722 -0.011527 0.011859 |
| PCA 6 -0.032834 0.055871 -0.043575 0.934732 0.018943 -0.343560 -0.042181 -0.002466 -0.000683 |
| PCA 7 0.043889 -0.115997 0.055398 -0.163719 0.879263 -0.426035 -0.008383 0.001509 0.008080 |
| PCA 8 0.271954 -0.733117 0.604277 0.093871 -0.117548 0.027659 -0.005069 0.002900 0.001335 |
| PCA 9 0.008712 -0.000941 -0.009163 0.000437 -0.009628 -0.001105 -0.000390 -0.000166 0.999872 |

**Table 4: Thematic layer Categorization of the controlling factors and their rank, weight affecting the mineralogical alteration, and radioelements potential evaluation.**

|  | **Thematic map** | **Classes** | **weight (100)** | **Rank** |
| --- | --- | --- | --- | --- |
| **1** | **Airborne eq. Uranium** | 1(140-170) | 20% | 5 |
|  |  | 2 (100-140) |  | 4 |
|  |  | 3 (50-100) |  | 3 |
|  |  | 4 (25-50) |  | 2 |
|  |  | 5 (0-25) |  | 1 |
| **2** | **Airborne eq. Thorium** | 1(140-170) | 10% | 5 |
|  |  | 2 (100-140) |  | 4 |
|  |  | 3 (50-100) |  | 3 |
|  |  | 4 (25-50) |  | 2 |
|  |  | 5 (0-25) |  | 1 |
| **3** | **Airborne eq. (K)** | 1(30-40) | 10% | 5 |
|  |  | 2 (20-30) |  | 4 |
|  |  | 3 (10-20) |  | 3 |
|  |  | 4 (5-10) |  | 2 |
|  |  | 5 (0-5) |  | 1 |
| **4** | **lineament density** | 1 | 15% | 1 |
|  |  | 2 |  | 2 |
|  |  | 3 |  | 3 |
|  |  | 4 |  | 4 |
|  |  | 5 |  | 5 |
| **5** | **geology** | younger and older Granite | 20% | 4 |
|  |  | Metamorphic rocks |  | 1 |
|  |  | Metasediments |  | 3 |
|  |  | Wadi Deposits |  | 4 |
|  |  | Ring complexes |  | 5 |
| **6** | **Anomaly and alteration map** | 1 (Hematite) | 25% | 3 |
|  |  | 2 (Kaolinite) |  | 4 |
|  |  | 3 (Illite- Sercite) |  | 5 |
|  |  | 4(Chlorite) |  | 2 |
|  |  | 5 (Hydroxyl) |  | 5 |
